# Functional Analysis of the Quorum-Sensing Streptococcal Invasion Locus (*sil*)

**DOI:** 10.1371/journal.ppat.1000651

**Published:** 2009-11-06

**Authors:** Ilia Belotserkovsky, Moshe Baruch, Asaf Peer, Eran Dov, Miriam Ravins, Inbal Mishalian, Merav Persky, Yoav Smith, Emanuel Hanski

**Affiliations:** 1 Department of Microbiology and Molecular Genetics, The Institute for Medical Research – Israel-Canada (IMRIC), The Hebrew University, Faculty of Medicine Jerusalem, Israel; 2 Genomic Data Analysis Unit of the Hebrew University Medical School, Jerusalem, Israel; Schepens Eye Research Institute, United States of America

## Abstract

Group A streptococcus (GAS) causes a wide variety of human diseases, and at the same time, GAS can also circulate without producing symptoms, similar to its close commensal relative, group G streptococcus (GGS). We previously identified, by transposon-tagged mutagenesis, the streptococcal invasion locus (*sil*). *sil* is a quorum-sensing regulated locus which is activated by the autoinducer peptide SilCR through the two-component system SilA-SilB. Here we characterize the DNA promoter region necessary for SilA-mediated activation. This site is composed of two direct repeats of 10 bp, separated by a spacer of 11 bp. Fusion of this site to *gfp* allowed us to systematically introduce single-base substitutions in the repeats region and to assess the relative contribution of various positions to promoter strength. We then developed an algorithm giving different weights to these positions, and performed a chromosome-wide bioinformatics search which was validated by transcriptome analysis. We identified 13 genes, mostly bacteriocin related, that are directly under the control of SilA. Having developed the ability to quantify SilCR signaling via GFP accumulation prompted us to search for GAS and GGS strains that sense and produce SilCR. While the majority of GAS strains lost *sil*, all GGS strains examined still possess the locus and ∼63% are able to respond to exogenously added SilCR. By triggering the autoinduction circle using a minute concentration of synthetic SilCR, we identified GAS and GGS strains that are capable of sensing and naturally producing SilCR, and showed that SilCR can be sensed across these streptococci species. These findings suggest that *sil* may be involved in colonization and establishment of commensal host-bacterial relationships.

## Author Summary

Cell-to-cell communication in bacteria is termed quorum-sensing (QS), which is triggered by signaling molecules called autoinducers. In streptococci, autoinducers are synthesized as immature peptides that are processed, secreted, and then sensed by two-component systems (TCSs). As a result, the autoinducer's own expression is upregulated (autoinduction), subsequently creating an ultrasensitive switch that turns on more genes. Group A streptococcus (GAS) is a human pathogen that causes many infections, including necrotizing fasciitis (NF). Previously, we identified in a NF GAS strain a QS locus termed streptococcal invasion locus (*sil*). Due to a mutation in the autoinducer peptide-SilCR, it is not produced by this strain. Here we sought to better explore *sil* and to examine if SilCR can be produced by other GAS strains, or strains of its close relative group G streptococcus (GGS). To this end, we characterized the DNA promoter region responsible for the TCS-mediated activation upon sensing of SilCR, and based on bioinformatics and transcriptome analyses we identified genes that are directly affected by the autoinducer peptide. By converting SilCR response to fluorescence production and turning on the autoinduction circle with minute concentrations of synthetic SilCR, we discovered naturally SilCR-producing GAS and GGS strains, and showed that SilCR can be sensed across these species. Our study describes a novel way of cell-to-cell communications among streptococci.

## Introduction

Group A streptococcus (GAS) is a common human pathogen that has major healthcare and economic impacts. It causes a variety of human diseases ranging from superficial skin and throat infections to severe life-threatening diseases such as necrotizing fasciitis (NF). There are approximately 10,000 cases of NF per year in the USA alone, estimated to cause 2400 deaths annually [1]. GAS is an exclusive human pathogen and many individuals carry GAS asymptomatically in their upper respiratory tract and other anatomic sites [2].

Little is known about what controls the conversion from a carrier to a pathogenic state in GAS infections. In fact, the distinction between commensals and pathogenic bacteria in general is blurred. This is mainly because both categories of bacteria use common modes of interaction with their hosts [3]. Both types of bacteria generally operate as communities and not as solitary microorganisms, hence, bacterial communication systems are key elements in host-bacterial interactions [4]. Bacteria communicate by secreting and subsequently sensing signal molecules [4],[5]. These molecules are termed autoinducers because when their concentration exceeds a given threshold, their own expression, as well as that of other genes, is abruptly activated [4],[5]. This activation usually occurs at high bacterial cell densities, which ensures that the increased gene expression only takes place in the presence of a sufficient number (a quorum) of cells, giving this mechanism the name quorum-sensing (QS.)

In Gram-positive bacteria autoinducers are synthesized as a pro-peptide that is processed and secreted by ATP-binding cassette (ABC) transporters. The mature peptide is then sensed by two-component signal transduction systems (TCSs). This triggers an up-regulated expression of the peptide itself (autoinduction) and often of the TCS and the ABC transporters, thus creating an ultrasensitive regulatory switch that subsequently leads to a change in the expression of an array of genes. These types of systems have been found to regulate various processes in streptococci including virulence, genetic competence, bacteriocin production, acid tolerance and biofilm formation [6],[7],[8],[9],[10].

**Figure 1:**
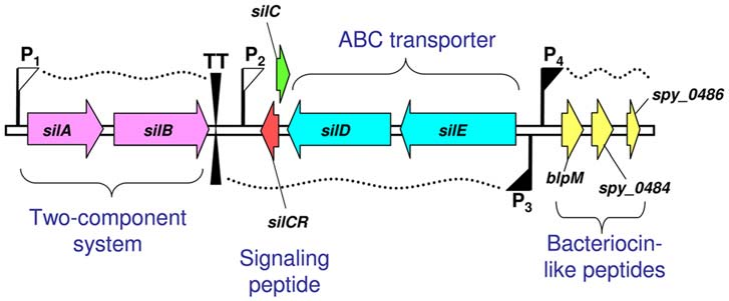
Transcriptional organization of the *sil* locus as previously deduced. *sil* is known to contain three polycistronic transcriptional units: *silA/B* encoding the TCS SilA-SilB, *silE/D/CR* encoding the ABC transporter system (SilD/E), plus the signaling peptide SilCR, and *blp* encoding the bacteriocin-like peptides BlpM, *spy_0484* and *spy_0486*. The transcription of the above-mentioned units is initiated from P_1_, P_3_, and P_4_ promoters, respectively. The monocistronic transcript of *silC* is initiated from the P_2_ promoter. Promoters induced and non-induced by SilCR are illustrated by filled and empty flags, respectively. Arrows indicate genes; waved-dotted lines represent transcripts; TT stands for a transcriptional terminator.

By applying transposon-tagged mutagenesis (STM) on a strain isolated from a NF patient (M14 type strain JS95) and using a murine model of human soft-tissue infections, we identified the streptococcal invasion locus (*sil*) [11]. Subsequent analysis showed that *sil* is composed of nine genes organized in four transcriptional units (Figure 1). A unit of a polycistronic mRNA, initiated from the P_1_ promoter, encodes a TCS SilA-SilB composed of a response regulator and a histidine kinase. An additional unit of polycistronic mRNA, initiated from the P_3_ promoter, which is transcribed in the opposite direction, encodes an ATP-binding cassette (ABC) transporter system SilD-SilE and the autoinducer peptide SilCR. However, in the NF strain JS95, SilCR is not formed due to a start codon mutation (ATG to ATA) [11],[12]. The third polycistronic mRNA unit, initiated from the P_4_ promoter, encodes bacteriocin-like peptides. Finally, the forth monocystronic mRNA initiated from the P_2_ promoter contains *silC* that highly overlaps *silCR* and is linked to the ability of GAS to spread in soft-tissues [11],[13]. The basal level of transcription from the P_3_ and P_4_ promoters is extremely low but abruptly increases when a mature synthetic SilCR peptide is added to the culture medium of JS95. The increased transcription of *silE/D/CR* is dependent on the dose of the added SilCR peptide and absolutely requires the TCS SilA-SilB [12].

We previously hypothesized that loss of SilCR and its regulatory potential might play a role in the highly invasive phenotype of JS95 and in the virulence of other GAS strains containing the *sil* locus [11],[14]. In line with this notion, all the M14-type isolates identified in a survey of invasive GAS infections in Israel [15], have a point mutation in the start codon of SilCR (unpublished data), and the M4 and M18-type GAS strains have a truncated *silD* [16].

*Streptococcus dysgalactiae* subsp. equisimilis contains several taxonomic units including group G streptococci (GGS) [17]. GGS are close genetic relatives of GAS, and are usually regarded as commensals [18], but recently were shown also to cause human invasive infections [19],[20]. GGS and GAS occupy similar niches in the host and can exchange genetic material [21],[22],[23],[24], which in streptococci usually requires sensing and communication across species [25]. Since *sil* has been suspected to be acquired by horizontal gene transfer [11], we also examined *sil* presence in GGS. Here we show that while *sil* is present only in a small fraction of GAS strains, its prevalence in GGS is much higher. We discovered that GAS and GGS strains naturally produce SilCR and demonstrate, for the first time, that the peptide can be sensed across these streptococci species.

## Results

### Identification of DNA sites necessary for SilA-mediated promoter activation

**Figure 2:**
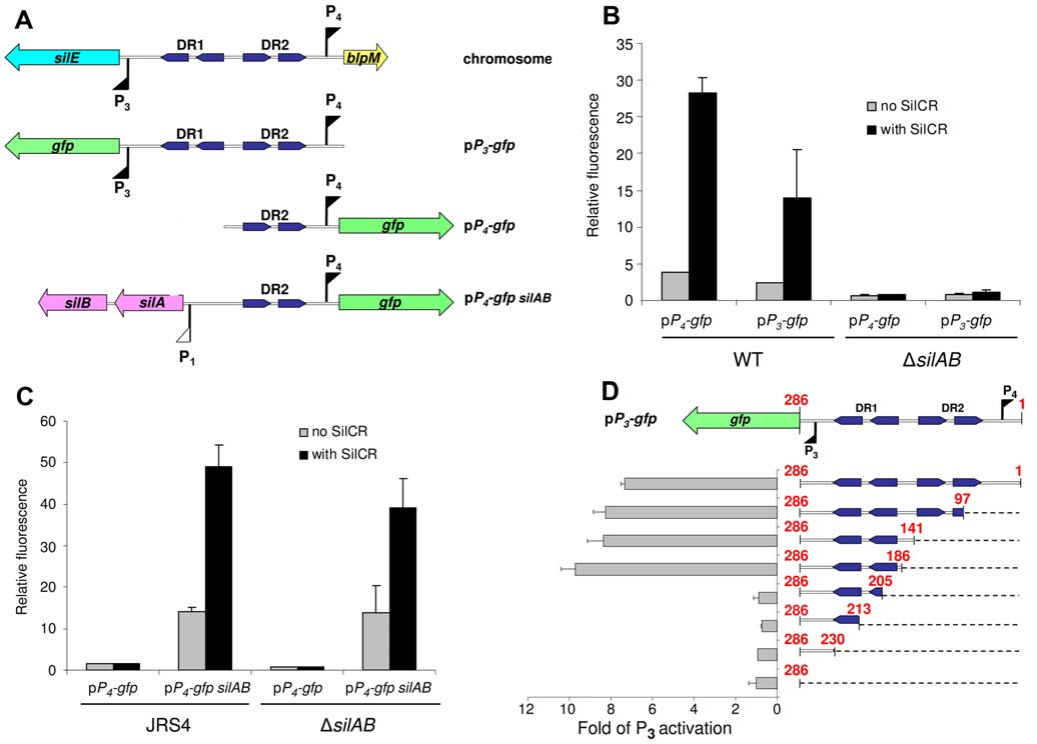
Identification of DNA motifs which serve as putative binding sites of SilA. (A) A schematic representation of the *silE-blpM* intergenic region, and the construction of reporter plasmids. The p*P_3_-gfp* and p*P_4_-gfp* plasmids were constructed by fusing P_3_ and P_4_ promoters, together with the entire intergenic region or part of it, to a promoterless *gfp* reporter. The p*P_4_-gfp silAB* plasmid consists of the original p*P_4_-gfp* with the addition of a DNA segment encoding the TCS SilA-SilB and its native promoter. Blue arrows indicate Direct Repeats (DR). Colored big arrows indicate genes and flags stand for SilCR-induced (filled flag) or non-induced (empty flag) promoters. (B) The activation of the P_3_ and P_4_ promoters by SilCR is absolutely dependent on the TCS SilA-SilB. JS95 WT and its derived mutant Δ*silAB* harboring either the p*P_4_-gfp* or the p*P_3_-gfp* plasmids were grown to an OD_600_ = 0.3 in the presence or absence of 10 µg ml^−1^ of SilCR. The fluorescence of the PBS-washed bacteria was measured by a fluorometer and normalized to the optical density. (C) The expression of the TCS SilA-SilB is necessary for transcription activation of P_4_ in a Δ*silAB* mutant and sufficient for activation of P_4_ in the *sil*-deficient M6-type GAS strain, JRS4. Relative fluorescence of the JRS4 and JS95-derived Δ*silAB* mutant harboring, either the p*P_4_-gfp* or p*P_4_-gfp silAB* plasmid, was determined as in (B). (D) Delineation of the minimal DNA segment required for P_3_ activation. A set of plasmids harboring different lengths of the *silE-blpM* intergenic region (right-hand side) was generated by progressively and unidirectionally digesting the DNA segment from P_4_ to P_3_ (see “Materials and Methods”). The fluorescence of JS95 strains harboring these plasmids was determined as in (B). The results are expressed as a fold of P_3_ activation calculated for each strain by dividing the normalized fluorescence values obtained upon their growth in the presence and absence of SilCR. The values shown in panels B, C and D represent the mean ± the standard deviations of at least two independent experiments.

We have previously shown that upon addition of synthetic SilCR peptide the P_3_ and the P_4_, promoters of *sil* (Figure 1) are stimulated in the GAS strain JS95 (Table S1), and this upregulation absolutely requires the TCS SilA-SilB [12]. SilA belongs to the AlgR/AgrA/LytR family of transcription regulators that bind DNA via a LytTR-type domain [26]. To activate promoters, these regulators form dimers and bind to two direct repeats [27],[28],[29],[30],[31],[32],[33],[34]. Indeed, upstream of the P_3_ and P_4_ promoters we identified two sets of direct imperfect repeats, which we termed DR1 and DR2, respectively (Figure 2A). To examine whether these repeats are involved in the activation of the P_3_ and P_4_ promoters, we fused the DNA segments depicted in Figure 2A to a promoterless *gfp* reporter and both elements were subsequently cloned into an *E. coli*-Gram-positive shuttle vector, yielding the plasmids p*P_3_-gfp* and p*P_4_-gfp* (Figure 2A). When transformed into the JS95 strain, both p*P_3_-gfp* and p*P_4_-gfp* produced the green fluorescent protein (GFP) only in the presence of synthetic SilCR (Figure 2B). The production of GFP by both promoters was absolutely dependent on intact SilA-SilB, since no GFP accumulation was observed in a JS95 mutant-deficient of the TCS (Figure 2B). These results support the notion that binding of SilCR to the histidine kinase sensor, SilB, leads to activation of the transcription regulator SilA, which in turn stimulates the P_3_ and P_4_ promoters. A dose-response relationship between the rate of GFP accumulation and SilCR concentration in GAS JS95 harboring p*P_4_-gfp* demonstrated that a maximal rate was reached at a concentration of approximately 5 ng ml^−1^ of SilCR (Figure S1A). To determine whether the SilA-SilB TCS is not only necessary but may be sufficient for the activation of the P_4_ promoter, we cloned the genes *silA* and *silB* together with their native promoter into the p*P_4_-gfp* plasmid, yielding the plasmid p*P_4_-gfp silAB* (Figure 2A and Table S2). When the plasmid was transformed into the JS95 mutant lacking SilA-SilB it complemented the SilCR-mediated GFP accumulation, demonstrating that SilA-SilB are expressed from the plasmid and are necessary for promoter activation (Figure 2C). JRS4 is an M6-type GAS strain which does not possess *sil*. Due to lack of SilA-SilB, transformation of JRS4 with p*P_4_-gfp* was insufficient for the production of GFP either in the presence or the absence of SilCR (Figure 2C). By contrast, JRS4 transformed with p*P_4_-gfp silAB* produced at least 3-fold more GFP in the presence of SilCR than in its absence, demonstrating that SilA-SilB can be functional also in a heterologous GAS background, and thus is also sufficient for P_4_ activation. It is apparent that a relatively high level of GFP was produced by the strain transformed with p*P_4_-gfp silAB* even in the absence of SilCR (Figure 2C). This probably results from an excess of SilA that is expressed from the plasmid. At such high concentrations, even an unphosphorylated response regulator might be able to bind to the DR2 site and partially activate the P_4_ promoter.

To define more accurately the DNA sequence necessary for activation of the P_3_ promoter by SilA, we deleted unidirectionally and progressively the 286 bp intergenic DNA segment located between *blpM* and *silE* from the direction of *blpM* (P_4_) towards *silE* (P_3_) (Figures 2D and S2). A deletion of the first 186 bp that included P_4_ and the DR2 site did not affect P_3_ activity, demonstrating that this site does not play a role in the stimulation of the P_3_ promoter (Figure 2D). By contrast, the DR1 site was absolutely necessary for P_3_ stimulation. Deletion of part of the distal repeat (205 out of the 286 nucleotides), completely abolished P_3_ activity, indicating that both repeats of DR1 are necessary to stimulate the transcription from the P_3_ promoter (Figure 2D).

**Figure 3:**
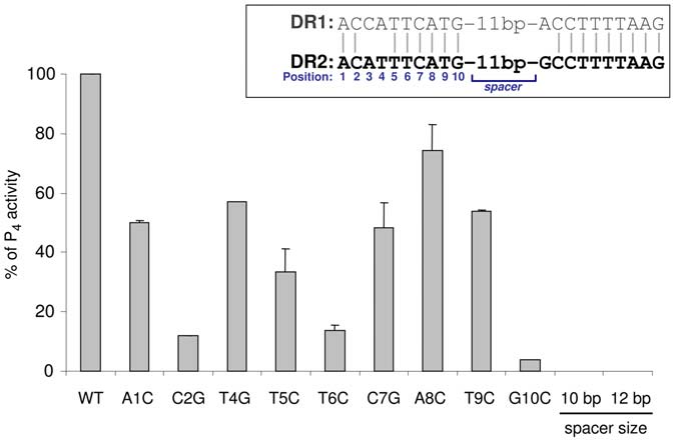
The relative contribution of the different positions in DR2 to the transcriptional strength of P_4_ promoter. The location of the positions and the invariant bases of DR1 and DR2 are shown in the inset. Substitutions of the indicated nucleotides with cytosine or guanine, and increase or decrease of the spacer size by a single nucleotide, were achieved by site-directed mutageneses that were performed on the p*P_4_-gfp* plasmid (see “Materials and Methods”). JS95 was transformed with p*P_4_-gfp* or with the resulting mutated plasmids. The corresponding strains were grown to an OD_600_ = 0.2, then SilCR was added to a final concentration of 10 µg ml^−1^ and the cultures were further incubated for 1 h. Fluorescence intensities were determined at time 0 and then every 15 min, as described in “Materials and Methods”. The slopes of fluorescence intensity as a function of time (representing initial rates of GFP accumulation) were calculated by performing least square analyses, which yielded coefficients of determination greater than 0.95 (R^2^>0.95). The P_4_ activity of JS95 transformed with non-mutated p*P_4_-gfp* (WT) was designated 100%, and the activities of the other mutants were calculated accordingly. The P_4_ activity in the absence of SilCR was negligible (not shown). The values shown are the mean±the standard deviation results of at least two independent experiments.

The consensus binding sequence of the LytTR domain is considered to consist of 9-bp repeats separated by 12 bp [26]. Since, in our case, the guanine nucleotide at position 10 is invariant in both DR1 and DR2 sites (Figure 3 inset), we decided to include it in the consensus sequence and evaluate the relative contribution of the various bases in the first repeat of DR2 to the P_4_ activity. This was achieved by systematically introducing single-base substitutions in 9 of the 10 nucleotides composing the first repeat of DR2 in p*P_4_-gfp*, forming a collection of plasmids harboring the desired mutations. Each plasmid was transformed into JS95 and the rates of GFP accumulation in the corresponding transformants were determined in comparison to that of the original p*P_4_-gfp* plasmid harboring the WT DNA segment. Substitution of the nucleotides at positions 2, 6 and 10 of DR2 had a marked detrimental effect on P_4_ activity, whereas the effect of substitutions at positions 1, 4, 5, 7, 8 and 9 was varied and less pronounced (Figure 3). To assess the importance of the spacing between the repeats of DR2 to P_4_ strength, we determined the effect of a nucleotide insertion (cytosine at position 16) or a nucleotide deletion (one of 6 adenines stretching between positions 14–19) as detailed above. Both modifications completely eliminated P_4_ activity (Figure 3), indicating that an exact spacing of the repeats is crucial for the SilA-dependent stimulation of the P_4_ promoter.

### Bioinformatics chromosomal-wide search for genes activated by SilA

**Figure 4:**
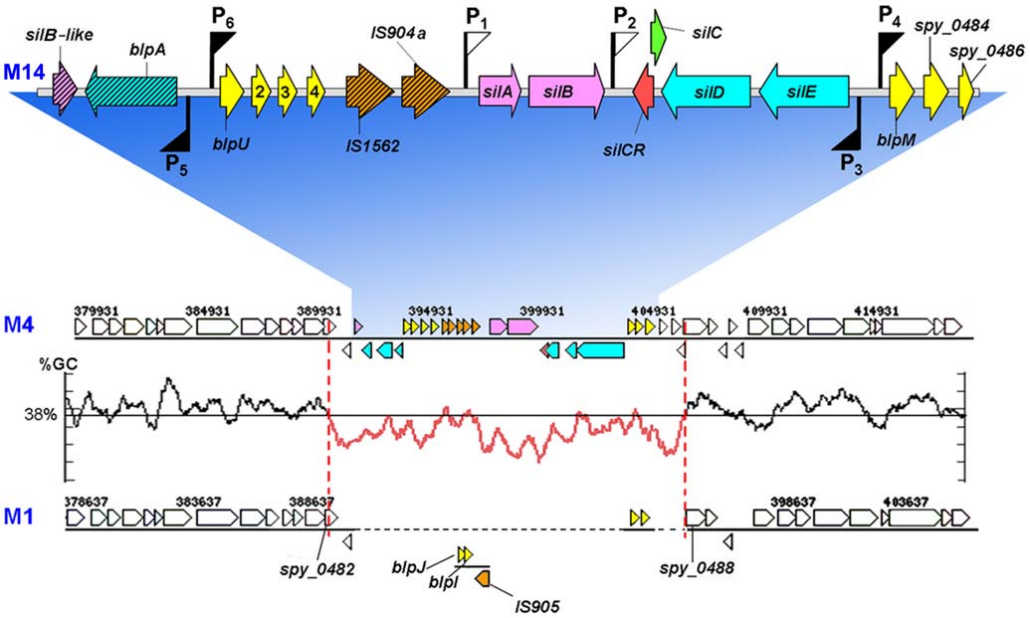
The transcriptional organization of the extended *sil* locus. The transcriptional organization and the gene content of *sil* as deduced from this study in the M14-type JS95 strain is enlarged (not to scale) (GenBank accession no GQ184568). Bold arrows indicate genes and flags represent promoters induced (filled) or non-induced (empty) by SilCR. Striped bold arrows symbolize pseudogenes. Key to colors: yellow, bacteriocin-like peptides; blue, ABC transporter systems; ochre, IS elements; pink, TCSs; red, signaling peptide. For gene alignment, ∼10 kb DNA segments located upstream and downstream of *sil* in the M4-type strain MGAS10750 containing *sil* (GenBank accession no NC_008024) were aligned with those of the *sil*-deficient M1-type strain SF370 (GenBank accession no NC_002737) using the Integrated Microbial Genomes (IMG) version 2.8 program [55]. The GC plot across *sil* in strain MGAS10750 was produced by the *freak* software (EMBOSS package [39]), using a stepping value of 100 and an averaging window of 500. The *blpI*, *blpJ* and *IS905* genes, present in the M1-type strain SF370, display no homology with *sil* from either MGAS10750 or JS95 strains, therefore these genes are positioned separately.

To identify SilA-regulated promoters we performed a bioinformatics search using a position specific probability matrix based on the sequences of DR1 and DR2 (Figure 3). Since the genome of the M14-type strain JS95 has not been sequenced yet, the search was performed on the genome of the M4-type strain MGAS10750 (GenBank accession no NC_008024), containing *sil* (Figure 4). First we used a probability matrix that assumed independence between the positions. However, since only two binding sites have been identified (DR1 and DR2), the probability matrix was overfitted. To overcome this drawback, we used the data obtained from the experiments that yielded numerical values for the contributions of the various positions in DR2 to promoter strength (Figure 3), and performed the computation analysis according to the algorithm detailed in “Materials and Methods”. The search identified two new putative SilA-binding sites located upstream to promoters P_5_ and P_6_ in a neighboring location to *sil* (Figure 4). Like P_3_ and P_4_ promoters, P_5_ and P_6_ initiate divergent transcription of an ABC transporter and bacteriocin–like peptides, respectively (Figure 4). The mRNA initiated from the P_6_ promoter includes 6 ORFs; of which the first 4 were shown by us to be situated on the same transcript (Figure S3A). As expected, their transcription was strongly stimulated by the presence of SilCR and was dependent on the TCS SilA-SilB (Figure S3B). The first ORF displays homology [∼60% amino acid (aa) identity] with the bacteriocin BlpU of *S. pneumoniae* [28]. Since, orthologuous genes are present in *Streptococcaceae* [30],[35],[36], we also designated this gene as *blpU*. ORF2 is an 80 aa-long protein that in its N terminal portion displays homology with a superfamily of membrane-bound CAAX metalloproteases [37]. This family of proteins has been implicated in protection against or maturation of bacteriocins [29]. The first 30 aa of ORF3 contain a signal leader peptide common to almost all *Streptococcal* bacteriocins. Although ORF4 contains a double-glycine motif required for processing of bacteriocins, it does not display homology to bacteriocin-related genes. The next two ORFs encode the insertion sequences *IS1562* and *IS904a*, but they are truncated (Figure 4). The divergent P_5_ promoter, containing a putative SilA-binding site, initiates the transcription of a bacteriocin transporter homologous to BlpA of *S. pneumoniae* [28]. This gene appears to be truncated and its transcription was moderately stimulated by SilCR, as described below (Table S5).

When *sil* was discovered, we found that it contains a lower GC content than the 38.5% average of the GAS chromosome [38]. Therefore, we suggested that *sil* might be a part of a genomic island [11]. To identify potential boundaries of the entire *sil* locus, in view of the adjacent positions of P_5_ and P_6_, we now calculated the percentage of G+C using the EMBOSS program *freak* [39] across *sil* in the M4 strain MGAS10750 (GenBank accession no NC_008024). We identified a sharp decline in the GC percentage around *spy_0482* (Figure 4). The region of low GC content stretches over 15.5 kbp and GC sharply increases to 38.5% around *spy_0488* (Figure 4). The good concurrence between the transcription studies and the GC content changes made us redefine *sil* as the entire 15.5 kbp region. Finally, sequencing of the region upstream to SilA in the M14 strain JS95 verified that the entire *sil* locus of JS95 (GenBank accession no GQ184568) is highly homologous to that of M4-type strain MGAS10750 (GenBank accession no NC_008024), as shown in Figure 4.

The prevalence of *sil* among GAS strains ranges between 12 to 18% [12],[16],[40]. Only two out of the 13 sequenced genomes, belonging to M4 and M18-types, contain *sil*. Nonetheless, all the other genomes possess *blpM*, *spy_0484* and *spy_0486*, which constitute an integral part of *sil* (Figure 4). Moreover, in all sequenced genomes, these genes are located in the same genomic location as in the M4-type *sil*-possessing strain, as shown for the *sil-*deficient M1-type SF370 strain (accession no NC_002737) in Figure 4. Intriguingly, GAS strains, which do not possess *sil*, still retain the bacteriocin-like genes *blpM* and *spy_0484*, considered to be a part of *sil*. Furthermore, they also have genes encoding bacteriocin-like peptides BlpI and BlpJ [28] (flanked by a part of the *IS905* transposon element) in the same location where *sil* would have been in these strains (Figure 4). This implies that due to their extensive sequence similarity, bacteriocins might serve as hotspots for genetic rearrangements.

### Chromosomal-wide search for genes whose transcription is affected by SilCR

**Figure 5:**
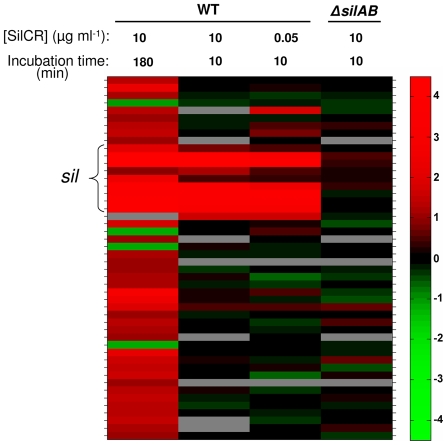
Global transcriptional analysis of JS95 in the presence of SilCR. To identify genes whose transcription is affected by SilCR, we exposed strain JS95 and its Δ*silAB* mutant to the conditions specified, and performed transcriptome analyses using a “universal” GAS microarray, as detailed in “Materials and Methods”. The results represent gene loci whose expression ratio changed by 2-fold or more with a p-value<0.05, at least in one of the experiments, visualized using the MatLab software. The genes are ordered vertically, based on their position in the genome (NC_008024) of M4-type strain MGAS10750 containing *sil*. The 9 genes of *sil* locus are indicated by a bracket. Grey bars represent cases when the replicate probes were highly inconsistent. For numerical values of the presented data and gene names see Table S5. The data is also available in NCBI Gene Expression Omnibus (GEO) (accession no GSE16961).

To identify all the genes directly and indirectly regulated by SilCR, we performed gene expression analyses using a microarray. As mentioned above, the genome of JS95 has not been sequenced yet, therefore we constructed, together with NimbleGen, a “universal” *S. pyogenes* array. This microarray covers all ORF's from 13 sequenced *S. pyogenes* strains as well as a number of ORF's from strain JS95. A detailed description of the design of this array is found in “Materials and Methods”. Since *sil* transcription regulation was previously studied under conditions in which JS95 was grown to an early log in the absence and presence of 10 µg ml^−1^ of SilCR [12], we first performed the microarray analysis under these conditions. It was found that in the presence of SilCR the transcription of 46 genes was altered by at least 2-fold; 4 of which were down-regulated [Figure 5, Table S5, and NCBI Gene Expression Omnibus (GEO) (accession no GSE16961)]. As expected from a previous study [12] and the data shown above, the transcription of genes initiated from the P_3_, P_4_ and P_6_ promoters was strongly up-regulated by SilCR (Figure 5 and Table S5). As mentioned above, the transcription of the truncated *blpA* which is initiated from the P_5_ promoter was moderately up-regulated (Figure 4 and Table S5). Surprisingly, the transcription of *silB* (promoter P_1_, Figures 1 and 4) was slightly up-regulated, an effect previously undetected in real-time RT-PCR studies [12]. There was also an increase in the transcription of 37 genes that do not belong to *sil* (Table S5). To test the immediate response to SilCR, we performed the expression analysis using RNA samples that were prepared 10 min after SilCR addition. This period of time is sufficient for maximum transcription from the P_3_ and P_4_ promoters [12]. We found that under these conditions only genes having predicted SilA-binding sites were up-regulated (Figure 5 and Table S5). The *spy_0416* gene located downstream of *blpM* (nomenclature of M1 GAS SF370 strain accession no NC_002737) was also up-regulated but this is probably due to generation of a long read-through transcript from the P_4_ promoter. To ascertain that the transcription under these conditions is solely regulated via the SilA-SilB TCS, we performed transcriptome analysis using the JS95Δ*silAB* mutant, and found that there were no significant changes in gene transcription in the presence and absence of SilCR (Figure 5 and Table S5). We also performed a genome-wide expression analysis at 50 ng ml^−1^ of SilCR, a concentration that is similar to that actually produced by GAS and GGS strains possessing a functional *sil* (see below). We found that besides *spy_0416* and *spy_0150* (nomenclature of M1 GAS SF370 strain accession no NC_002737) only *sil* genes were affected (Figure 5 and Table S5).

These results taken together show that exposure of a *sil*-proficient GAS strain for a short time period to SilCR concentrations ranging from 50 ng ml^−1^ to 10 µg ml^−1^, mainly affects *sil* genes. However, prolonged incubation alters also the transcription of genes that do not possess the proposed SilA-binding sites (Figure 5, Table S5). To determine whether or not the change in these genes is SilA-mediated or is independent of SilA, requires additional analyses. Nonetheless, the fact that SilCR alters gene expression in a *sil*-deficient strain was reported by others [41]. In agreement with this observation, we found that *sagA* transcription was increased 4-fold when a Δ*silAB* mutant was grown to an early log phase (and not for 10 min as shown in Figure 5) in the presence of 10 µg ml^−1^ of SilCR (not shown).

### GAS and GGS strains are capable of producing and sensing SilCR

**Figure 6:**
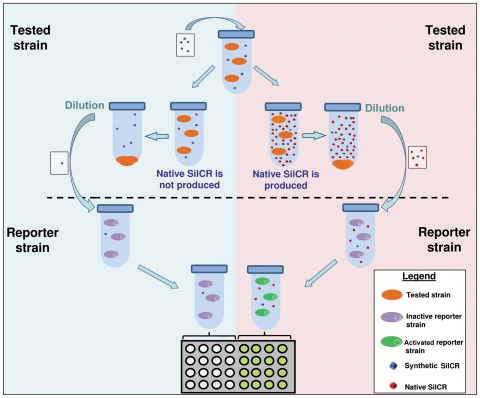
“Jump start” experiment scheme. The tested strain is grown in the presence of 5 ng ml^−1^ of synthetic SilCR to late log phase (OD_600_ = 1). Then, the supernatant of the tested culture is collected, diluted 10 or 3-fold and added to the “reporter strain” JS95 or N9 (respectively), harboring p*P_4_-gfp*. After incubation for 2 additional hours at 37°C, the relative fluorescence of the “reporter strain” is determined as described in Figure 2.

We previously reported, using a dot blot assay based on anti-SilCR antibody, that GAS is able to produce SilCR [12]. Yet, a more careful examination showed that this assay is prone to generating false positive results. To overcome this drawback, we used the ability of JS95 transformed with p*P_4_-gfp* to detect SilCR in the nanomolar range (Figure S1A), and searched for natively produced SilCR in the growth medium of different GAS strains. However, our attempts to trigger SilCR production by applying the stress conditions that induced competence in *S. pneumoniae* [42],[43], were unsuccessful. We therefore decided to switch on the autoinduction circle by providing *sil*-possessing strains with minute concentrations of SilCR, according to the strategy that is illustrated in Figure 6, and designated here as a “Jump start” experiment. To do so, tested strains were exposed to 5 ng ml^−1^ of synthetic SilCR. After 3 h of incubation, the bacteria were removed by centrifugation and the supernatant was collected and diluted 10-fold into the culture medium of JS95 transformed with p*P_4_-gfp*, which served as the reporter strain (Figure 6). If a tested strain is able to produce its own SilCR, the latter will accumulate in the culture medium and then induce GFP production in the reporter strain (Figure 6 right panel). If, however, the tested strain does not produce SilCR, the dilution of the supernatant medium prior to its addition to the reporter strain reduces the synthetic SilCR to a concentration that barely triggers GFP production (Figure 6, left panel, Figure S1A).

**Figure 7:**
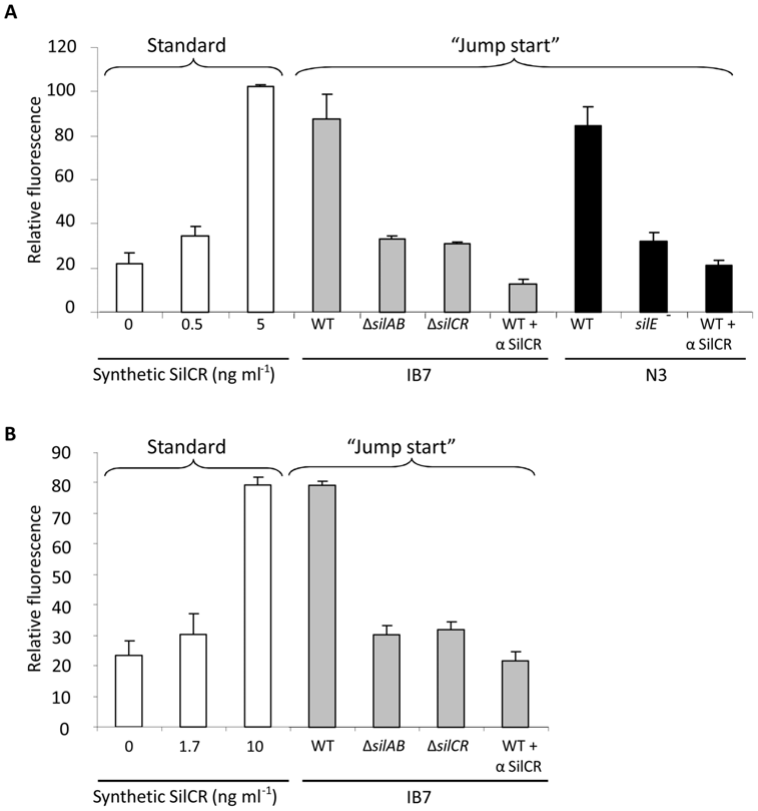
SilCR production and sensing by GAS and GGS. (A) GAS can sense SilCR produced by GAS IB7 and GGS N3. Relative fluorescence was determined for the JS95p*P_4_-gfp* “reporter strain” when grown with synthetic SilCR at the indicated concentrations (“Standard”, white bars) or in the presence of 10-fold diluted supernatants (“Jump start”) from IB7 (grey bars), N3 (black bars) and their derived mutants. α SilCR denotes addition of serum containing anti-SilCR polyclonal antibody [12], diluted 1∶40 into the reporter strain tube immediately before addition of the 10-fold diluted supernatants of the tested strains. The values shown are the mean ± the standard deviation results of at least two independent experiments. (B) GGS can sense SilCR produced by GAS IB7. Relative fluorescence of the N9p*P_4_-gfp* “reporter strain” when grown with synthetic SilCR at the indicated concentrations (“Standard”, white bars) or in the presence of 3-fold diluted supernatants (“Jump start”) from IB7 WT and Δ*silAB*, Δ*silCR* mutants (grey bars).

By applying this strategy, we found that GAS strain IB7 naturally produces SilCR. A 10-fold dilution of WT IB7 supernatant triggered GFP production in the JS95p*P_4_-gfp* strain to a relative fluorescence (RF) value that was comparable to that obtained by 5 ng ml^−1^ of synthetic SilCR (Figure 7A, white and grey bars). Mutants of IB7, deficient of either *silCR* or *silAB*, did not induce GFP production above the RF value produced by 0.5 ng ml^−1^ of synthetic SilCR. Moreover, when anti-SilCR antibody was added to the 10-fold diluted supernatant of IB7 WT strain, it completely blocked GFP production (Figure 7A, white and grey bars). This antibody captures the SilCR peptide and neutralizes GFP-production in a dose-dependent manner (Figure S4).

Since GAS and GGS are close relatives and were shown to exchange DNA elements [22], we screened several GGS isolates for *sil*. In contrast to ∼20% prevalence in GAS [12],[16], all the 12 GGS isolates tested possessed the locus as determined by PCR analysis (Table S6). In addition, 5 out of 8 (63%) GGS isolates tested responded to exogenously added SilCR when transformed with p*P_4_-gfp* (Table S6), while only 4 out of 14 *sil*-containing GAS strains [JS95, IB7, T13 and T14 (Table S1)] were positive in this assay. All examined GGS isolates contained ATG and not ATA as a start codon of SilCR but only one out of the 5 examined had an un-truncated *silD* (Table S6). Finally, we checked the ability of GGS isolates to produce SilCR using the “Jump start” assay described above. Among all isolates tested, only N3 (possessing an intact *silD*) was able to produce SilCR (Figure 7A, black bars). This ability was abolished when *silE* was interrupted by insertion-inactivation or when anti-SilCR antibodies were added to the reporter strain together with the supernatant of N3 WT (Figure 7A, black bars).

These data do not only suggest that GGS is capable of producing mature SilCR, but also that this peptide can be sensed by GAS. To demonstrate that the SilCR-mediated interspecies communication works in both directions, we performed a reciprocal experiment using GGS N9 harboring p*P_4_-gfp* as a reporter strain. Due to reduced (5 to 6-fold) sensitivity of this reporter strain compared to that of JS95 transformed with p*P_4_-gfp* (Figure S1A,B), we diluted the supernatant of the tested strain only 3-fold. A diluted supernatant of WT IB7 triggered GFP production in the N9 reporter strain to a RF value that was comparable to that produced by 10 ng ml^−1^ of synthetic SilCR (Figure 7B). In contrast, supernatants of Δ*silCR* or Δ*silAB* mutants of IB7, resulted in RF values equivalent to that produced by 1.7 ng ml^−1^ of synthetic SilCR, demonstrating that the mutants lost their ability to produce native SilCR. As shown above, anti-SilCR antibody reduced the GFP production to the background level. Taken together these results clearly show a bi-directional communication between GAS and GGS strains harboring functional *sil*.

Since IB7 and N3 were positive in “Jump start” experiments, we sequenced the core region of *sil* stretching from *silA* to *blpM* in both strains (GeneBank accession no, GQ184566 and GQ184567, respectively) and compared them to JS95 *sil* (accession no, GQ184568). As expected, *silCR* of IB7 and N3 contains ATG and not ATA as a start codon. Furthermore, these strains have an un-truncated *silD*. Since other GGS strains that could not produce SilCR possess ATG as the *silCR* start codon but have a truncated *silD* (Table S6), this strongly suggests that the ABC transporter system SilD-SilE is responsible for SilCR processing and secretion.

## Discussion

In this study we characterized in details the streptococcal invasion locus *sil* in GAS and GGS and showed, for the first time, that SilCR can be sensed across these streptococci species.

*sil* resembles the two separate regulatory networks that use different but paralogous TCSs to trigger competence (Com) and bacteriocin production (Blp) in the mitis group, (*S. pneumoniae*, *S. gordonii*, and *S. sanguis*) [44]. Although the organization of the *sil* locus is more similar to that of the Blp system, and SilA-SilB is more closely related to BlpR-BlpH, SilCR belongs to the family of competence-stimulating peptides [11].

Like many response regulators linked to autoinducing peptides, SilA belongs to the LytTR family [26]. Response regulators containing LytTR are known to dimerize and bind to 9-bp repeats, which are separated by a 12 bp spacer [27],[28],[29],[30],[31],[32],[33],[34]. We, however, found that the DNA element responsible for SilA-dependent activation of transcription is composed of two 10 bp repeats separated by a spacer of 11 AT rich bp. Recently a crystal structure of the LytTR domain of *S. aureus* AgrA bound to a 15 bp double stranded DNA was solved at a 1.6 Å resolution. It was shown that a side chain atom of LytTR forms contact with a nucleotide located at position 10 of the repeat [45], thus this finding supports our notion that position 10 in the DNA repeat plays an important role in SilA-mediated activation of the P_4_ *sil* promoter.

The bioinformatics and genome-wide transcription analyses performed here indicate that *sil* acts as an autonomous unit and contains almost all the genetic information necessary for its regulation by a QS mechanism. It is not known what controls the P_1_ promoter initiating the transcription of the *silA/B* TCS, however recent work shows that it is upregulated within the host [13]. The finding that *sil* is an autonomous unit supports the hypothesis that *sil* might represent an ancient mobile island that may have been acquired before GAS speciation. In GAS but not in GGS, subsequently a DNA segment stretching from the 5′ end of *sil* up to *silE* was lost from most strains. The presence of two truncated transposases (*IS1562* and *IS904a*) within *sil*, a truncated *silB* gene located at the 5′ border of *sil*, and the fact that the truncated *blpA* gene is highly homologous to *silE* (84% identical amino acids) may indicate that this location underwent an extensive genomic rearrangement. Furthermore, all GAS genomes that lost *sil* contain the same DNA segment, encoding the two bacteriocins, BlpI and BlpJ [28], flanked by a part of the *IS905* transposon, which is located at the same genomic position (Figure 4). This suggests that a second recombination event may have been responsible for *sil* excision, although other scenarios can not currently be ruled out.

Since most GAS isolates do not possess functional *sil* during invasive and rapidly disseminating infections, it is possible that *sil* might play a role during colonization or at the early steps of disease progress. Having developed the ability to quantify SilCR signaling via GFP accumulation, we show here that SilCR can be produced by some GAS and GGS strains and sensed across these species. This could have an important impact for establishing colonization. Our finding that 2 out of 4 transcriptional units that are upregulated by SilCR encode bacteriocins supports this notion even more, because these peptides control bacterial growth [46] and could be instrumental in cases of competition in that stage. A conclusion that only a minor portion of GAS and GGS strains possess a functional *sil* may be premature because, the current as well as most previous studies, were performed on GAS and GGS strains isolated from severe diseases [12],[15],[16],[40],[47]. Thus, it will be intriguing to explore the prevalence and the role of *sil* among GAS and GGS strains from carriers in which a commensal host-bacterial relationship is established.

## Materials and Methods

### Strains and media

All GAS and GGS strains used in the study are described in Table S1. For molecular cloning we used *Escherichia coli* strain JM109, which was cultured in Laria-Bertani broth, Lennox (Becton, Dickinson, and Sparks, MD, USA). GAS and GGS were cultured in Todd-Hewitt medium (Becton, Dickinson) supplemented with 0.2% yeast extract (Becton, Dickinson) (THY media) at 37°C in sealed tubes without agitation. To produce solid media, Bacto™ Agar (Becton, Dickinson) was added to a final concentration of 1.4%. When necessary, antibiotics were added at the following concentrations: for GAS and GGS: 250 µg ml^−1^ kanamycin (Km), 50 µg ml^−1^ spectinomycin (Spec) and 1 µg ml^−1^ erythromycin (Erm); for *E. coli*: 100 µg ml^−1^ ampicillin (Amp), 50 µg ml^−1^ Spec, 750 µg ml^−1^ Erm and 50 µg ml^−1^ Km. All the antibiotics were purchased from Sigma-Aldrich (St Louis, MO, USA).

### DNA manipulations

Plasmids and primers used in this study are listed in Table S2 and Table S3, respectively. The primers were synthesized by Syntezza Bioscience (Jerusalem, Israel). Plasmid DNA was isolated by commercial kits (Wizard®Plus Minipreps, Promega, Madison WI, USA), according to the manufacturer's instructions and used to transform *E. coli*, GAS and GGS strains by electroporation as described previously [48]. Restriction endonucleases, ligases and polymerases were used according to the recommendations of the manufacturers. Chromosomal DNA was purified from GAS as described previously [48] or by using the Wizard® Genomic DNA Purification Kit (Promega, Madison WI, USA). Linear DNA fragments were purified using Certified™ low-melt agarose (Bio-Rad, Hercules, CA, USA). PCR products were purified using a commercial kit (QIAquick PCR Purification Kit, (Qiagen, Hilden, Germany). DNA sequencing was performed using the Excel II cycle sequencing kit (Department of Genome Technologies, Givat Ram, The Hebrew University, Jerusalem, Israel). All other procedures were conducted according to standard protocols [49].

### Construction of mutants

#### (A) Deletion mutants

For construction of the Δ*silAB* mutant in the JS95 and IB7 strains, a 2222 bp DNA segment containing *silA* and *silB* was PCR amplified using the primers *silA*-f and *silB*-r (Table S3), and cloned into pGEM-T-Easy (Promega, Madison WI, USA), yielding the plasmid pG*silAB* (Table S2). A 1394 bp long segment from within *silA* and *silB* was excised by digesting pG*silAB* with *ClaI* and *BsrGI*, and replaced with the omega kanamycin cassette (ΩKm) [50], yielding pG*silAB*-ΩKm (Table S2). In this plasmid ΩKm is flanked upstream and downstream with residual ∼500 bp segments from the 5′ and 3′ ends of *silA* and *silB*, respectively. The entire insert was then released with *NotI* and cloned into the temperature-sensitive plasmid pJRS233 [51] digested with the same enzyme. The resulting plasmid pJ*silAB*-ΩKm was transformed into the JS95 and IB7 strains, and mutants were selected as previously described [51]. The deletion in each mutant was confirmed by sequencing a PCR fragment containing the junction regions between the chromosomal and inserted DNA fragment. Construction of IB7Δ*silCR* was performed as previously described for the JS95Δ*silC* mutant [11].

#### (B) Insertion inactivation mutant

The GGS N3*silE^−^* mutant was constructed using a pJ*silE* plasmid as previously described [12].

#### (C) Reporter plasmids

To construct p*P_3_-gfp*, the 233 bp intergenic *silE*-*blpM* region was PCR amplified using the primers *silEp*-f and *silEp*-r (Table S3 and Figure S2) that introduce *NsiI* and *StuI* sites at the 5′- and the 3′ ends, respectively. The PCR fragment was digested by these enzymes and subsequently cloned into the pKSM 410 *E. coli*-Gram positive shuttle plasmid harboring a promoterless *gfp* [52], digested with the same enzymes. To construct p*P_4_-gfp*, a 154 bp fragment located upstream to *blpM* (Figure S2) was amplified by PCR, using *blpMp*-f and *blpMp*-r primers (Table S3) that introduced *NsiI* and *XhoI* sites at the 5′- and the 3′ ends, respectively. This fragment was cloned into pKSM 410 plasmid that was digested with the same enzymes. To construct p*P_4_-gfp silAB*, *silA* and *silB* together with their native promoter P_1_ and transcriptional terminator (Figure 1) were PCR amplified using the primers *silA*-f and *silB* TT-r (Table S3), and the amplified fragment of 2565 bp was cloned into *SnaBI*-digested p*P_4_-gfp*.

#### (D) Reporter plasmids with different lengths of *silE-blpM* intergenic region

To define the minimal DNA segment required for SilA-mediated activation of P_3_, a library of plasmids containing fragments of different lengths from the intergenic *silE-blpM* region were generated. This was performed with the “Erase a Base” kit (Promega, Madison USA) according to manufacturer's protocol. Briefly, an exonuclease III sensitive blunt end was generated by digesting p*P_3_-gfp* with *StuI* (near P_4_). Then, an exonuclease III non-sensitive 3′ overhang end was created by *NspI*-digest, that also destroyed Spec-resistance in p*P_3_-gfp*. The *silE-blpM* intergenic DNA segment was progressively digested towards P_3_ with exonuclease III, upon incubating the reaction mixture at 4°C for short times ranging from 30 to 90 seconds. The lengths of the remaining undigested DNA segments in the resulting *E. coli* transformants were examined by colony PCR, using a fluorescently-labeled primer with FAM, followed by separation of the labeled products on a sequencing gel. To this end, we used the *gfp*-r and FAM-*spec*-r primers (Table S3), and the length of the PCR products was determined using ABI 3700 DNA Analyzer sequencer and Genescan® Analysis Software (Applied Biosystems, USA). The colonies harboring plasmids with the desired fragment lengths were chosen for further use.

#### (E) Site-directed mutagenesis

To construct single-base substitutions, addition or deletion mutations, we used the QuikChange® II XL Site-Directed Mutagenesis Kit (Stratagene, La Jolla, CA USA) according to manufacturer's protocol. The p*P_4_-gfp* reporter plasmid served as a template for point mutations using primers containing the desired base substitutions as described in Table S3. All reporter plasmids described in (C) (D) (E) were introduced into GAS or GGS strains (Table S1) by electroporation and transformants resistant to Spec and/or Km were isolated.

### RNA isolation

RNA was purified by applying one of two procedures. JS95 or its derivative mutants were grown in THY to OD_600_ = 0.2, the cultures were divided among several tubes and the desired amount of synthetic 96% pure SilCR (BioSight, Israel) was added to each tube, which was then incubated for 10 min at 37°C. Alternatively, bacteria were grown to the desired growth phase with or without 10 µg ml^−1^ of SilCR that was added at the beginning of culture growth. Total RNA from bacteria was isolated by hot acidic phenol extraction as previously described [53]. For microarray analysis the RNA concentration and integrity was verified by capillary gel electrophoresis in the Agilent 2100 Bioanalyzer. Only samples with an RNA integrity number equal or higher than 8 were used.

### Real time RT-PCR

RNA (4 µg) was treated with RQ1 DNase (Promega, Madison WI, USA) and subjected to cDNA synthesis using MMLV reverse transcriptase (Promega, Madison WI, USA), according to the manufacturer's protocols and as described previously [12]. Standard real time RT-PCR reactions were conducted using. SYBR-green mix (Absolute SYBR GREEN ROX MIX, ABgene) and fluorescence detection was performed using Rotor-Gene 3000 A (Corbett life Science, Qiagen Germany GmbH) according to manufacturer's instructions. RT-PCR primers (Table S3) were designed using Primer Express™ software v2.0 (Applied Biosystems). The cDNA amount of gyrase subunit A (*gyrA*) was used to normalize expression data for each target gene. Each assay was performed in duplicates with at least two RNA templates prepared from independent bacterial cultures grown on different days. The data were analyzed according to the standard curve method (Rotor-gene analysis software 6.0) and are presented as abundance of transcript amount relative to that of *gyrA*.

### Fluorescence measurements

Bacteria harboring plasmids containing *gfp* (Table S2) were grown and treated as described in the text, washed twice with phosphate-buffered saline (PBS), concentrated 10-fold by resuspension in PBS at 1∶10 of the original volume and transferred in duplicates of 0.3 ml into 96-well flat bottom transparent plates (FluoroNunc™). The relative fluorescence data were measured in a fluorescence/absorbance reader Infinite F200 (Tecan, Austria GmbH) using the filter set 485/20 nm for excitation, 535/25 nm for emission. The fluorescence data were normalized according to the density of the cultures, which was determined by measuring OD at 595 nm. For simplicity the relative fluorescence values were divided by 100 for presentation.

To measure promoter strength, the initial rates of GFP accumulation were calculated. For this purpose, cultures were grown to an OD_600_ = 0.2 and then various concentrations of synthetic SilCR were added. Fluorescence intensity was determined for time 0 and then for 4 additional time points, using samples withdrawn every 15 min. Promoter activity was calculated from slopes of fluorescence intensity as a function of time by performing least square analyses which yielded coefficients of determination greater than 0.95 (R^2^>0.95).

### “Jump start” experiment

For the “Jump start” experiments strains were grown in THY supplemented with 10% (V/V) of fetal bovine serum (Biological Industries, Beit Haemek, Israel) in the presence of 5 ng ml^−1^ of SilCR until late log phase (OD_600_ = 1). Then, the cultures were centrifuged for 10 min at 14,000 g and the supernatants were diluted 10 or 3-fold into the culture media of JS95p*P_4_-gfp* or N9p*P_4_-gfp* (respectively) which served as “reporter strains”. Relative fluorescence of the “reporter strain” was determined after an additional incubation of 2 h at 37°C, as described above.

### Bioinformatics search for SilA-binding sites

For the bioinformatics search for SilA-binding sites we used a probability matrix *(M)*. This matrix assumes independence between the different positions of the binding site, and accordingly the probability 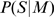 is 
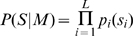
 where *L* is the length of the binding site and 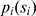 is the probability to find a specific nucleotide in position *i*. The probability matrix that takes into consideration the relative weights of the positions in the binding site motif is 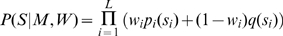, where 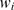 represents the weight assigned to each position of the motif; 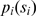 is as explained-above and 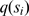 is the frequency of the nucleotide in the genome. For the search over the genome of M4-type strain MGAS10750 containing *sil* (GenBank accession no NC_008024) *q*(cytosine) and *q*(guanine) were designated as 0.192 and *q*(adenine) and *q*(thymine) were designated as 0.308. The relative weights of the positions in the binding site motif were based on the results of the point mutations in DR2, shown in Figure 3. For each position 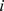, 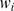 was determined as 1 minus the ratio of P_4_ activity for the specific mutant to that of the WT promoter. Although the mutation experiments were performed only on the first repeat, the weights were assumed to be suitable also for the second one. Mutating position 3 of the binding site was not successful, therefore 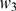 was assumed to be equal to 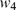. The parameters of the two motifs are given in the Table 1. Assigning small values to *w_i_* can result in a deprecated algorithm, therefore we also verified that the most probable binding sites found are not coincidental by computing a p value based on a comparison to random sequences (see Table S4).

**Table 1:** The parameters used for the computational predictions.

Position in (repeat 1)	A	C	G	T	Weight
1	1	0	0	0	0.5
2	0	1	0	0	0.9
3	0.5	0.5	0	0	0.5
4	0.5	0	0	0.5	0.5
5	0	0	0	1	0.66
6	0	0	0	1	0.87
7	0	1	0	0	0.52
8	1	0	0	0	0.25
9	0	0	0	1	0.42
10	0	0	1	0	0.96

Position in (repeat 2)	A	C	G	T	Weight
1	0.5	0	0.5	0	0.5
2	0	1	0	0	0.9
3	0	1	0	0	0.5
4	0	0	0	1	0.5
5	0	0	0	1	0.66
6	0	0	0	1	0.87
7	0	0	0	1	0.52
8	1	0	0	0	0.25
9	1	0	0	0	0.42
10	0	0	1	0	0.96

The parameters assigned for nucleotide abundance and the weights of the different positions in the repeats are indicated in the table and explained in the text.

Using this model, the genome was screened for putative SilA binding sites by computing the probability of finding the two direct repeat motifs with a spacer of 11 nucleotides, starting at each position of the genome. The probabilities of finding each of the two repeats were multiplied and their natural log was computed and used as a score for comparing the different binding sites found. The statistical significance of each score was obtained by its comparison to scores of random sequences. We extracted a million random subsequences of 31 nucleotides (the size of the entire binding site) from the query, shuffled each of them and computed the score of each shuffled sequence. For each candidate binding site in the original genome sequence, the p-value of its score was obtained by the fraction of random 31-mers that exceeded this score. The candidate sites with a p-values lower than 10^−5^ were considered for further analysis and can be found in Table S4.

### Microarray

#### (A) Design, cDNA labeling, hybridization and scanning

To enable study of gene expression profiles for non-sequenced GAS strains, we designed, together with Roch NimbleGen Systems GmbH, a “universal” GAS expression array. The design of this array is based on the identification of unique ORFs. An ORF is considered unique when it differs from homologous ORFs by even a single nucleotide. This resulted in 17,047 unique ORFs, identified by screening 12 GeneBank available GAS genomes: (NC_002737, NC_008022, NC_006086, NC_008024, NC_008023, NC_004070, NC_007297, NC_007296, NC_003485, NC_008021, NC_004606, NC_009332), and the partially annotated chromosome of M49-type strain 591. A number of sequenced ORFs from the M14-type strain JS95 were added as controls. Probes could not be designed for 306 unique ORFs mainly due to low complexity sequences. The remaining 16,741 ORFs are represented by 9299 exemplars (i.e. distinct probe sets), 16,565 of them are covered by 7 probes and 176 are covered by 1 to 6 probes. Altogether, the array contains 64318 60-mer experimental probes as well as a number of internal quality controls.

Using this array we performed two-color cDNA experiments and compared relative levels of gene expression. cDNA was produced with random hexamer primers (6 µg) from 2 µg of total RNA using the superscript III reverse transcriptase (Invitrogen, Carlsbad, CA, USA). The reverse transcription reaction was carried out at 42°C for 16 h with aminoallyl (aa) labeled dUTP. Unincorporated aa-dUTP and free amines were removed using Microcon YM-30 filters (Millipore, Billerica, MA, USA) according to the manufacturer's recommendations. Coupling of aminoallyl labeled cDNA to Cy-dye esters was performed in 0.1 M sodium carbonate buffer (pH = 8.6) for 1 h at room temperature. Free dye was then removed from the samples using the Qiagen QIAquick PCR purification columns, after which hybridization efficiency was measured using a spectrophotometer (ND-100, Nanodrop Technologies), and labeled cDNA concentration was calculated. Cy3-labeled sample (1 µg) was mixed with an equal amount of Cy5-labeled sample, and the mixture was dried using a DNA concentrator. Hybridization was performed according to NimbleGen protocol (NimbleChip™ arrays user's guide for gene expression analysis) at 42°C for 16 h using the NimbleGen MAUI hybridization system. Hybridized arrays were washed, and the slides were scanned using Axon GenePix 4000B scanner (Axon Instruments) with settings adjusted to obtain similar green and red intensities. Scanned images were imported separately for each color (wavelength 532 nm for Cy3 and 635 nm for Cy5) into NimbleGen's NimbleScan software (Nimblescan™ 2.3). Using the software each image was gridded and a pair report was created containing the raw data listing the intensity of each probe.

#### (B) Expression analysis

The two pair reports of each two-color experiment were imported into a specially written MatLab (R2007b) program. The data underwent a process of flooring (expression data of less than 20 was assigned a value of 20). Then, a scatter plot was created, plotting log10 of the product of the Data X and Data Y intensities against log2 of their intensity ratios. This was subjected to Lowess normalization method using a linear fit with a window of 0.05 (5%). Since for most of the ORFs there are 7 probes the data was reshaped so that each row contained all the data per each ORF (7 readings of CY3 followed by 7 readings of CY5). At this point a t-test was performed on each row and a p-value was calculated using 1000 permutations. The log2 ratios of the average 7 readings of CY5 divided by the average of 7 readings of CY3 was calculated for each ORF row. The ORF data was then presented in a Volcano plot using the vertical axis for the p-values and the horizontal axis for the log2 ratio values. By using a threshold of less than 0.05 for the p-values, and a fold change of + or − 2 for the absolute log2 ratios, ORFs with the largest statistical significant expression change could be selected. The data was submitted to NCBI Gene Expression Omnibus (GEO) accession no GSE16961.

### Sequence analysis

Searches for homologuos genes and proteins were performed using BLASTN and BLASTP [54] 2.2.20 programs against non-redundant databases with default parameters. Genomic regions alignments were performed using Integrated Microbial Genomes (IMG) program version 2.8 [55]. The GC plot was produced by *freak* software (EMBOSS [39]) using a stepping value of 100 and an averaging window of 500.

### List of accession numbers

12 GeneBank available GAS genomes: NC_002737, NC_008022, NC_006086, NC_008024, NC_008023, NC_004070, NC_007297, NC_007296, NC_003485, NC_008021, NC_004606, NC_009332.

GeneBank accession no of IB7 *sil* locus - GQ184566.

GeneBank accession no of N3 *sil* locus - GQ184567.

GeneBank accession no of JS95 *sil* locus - GQ184568.

Microarray data - NCBI Gene Expression Omnibus (GEO) accession no GSE1696.

## Supporting Information

Table S1 Strains
(0.07 MB DOC)

Table S2 Plasmids
(0.04 MB DOC)

Table S3 Primers
(0.07 MB DOC)

Table S4 Predicted SilA binding sites and their genetic context
(0.05 MB DOC)

Table S5 Microarray analysis
(0.10 MB DOC)

Table S6 Characterization of *sil* in GGS isolates. *sil* presence was verified by PCR using the SP-02 and SP-04 primers. The *silCR* start codon and the intactness of *silD* were tested by amplifying the corresponding regions by PCR using the SP-02 and SP-04, and *sil*E-04-r and ABC-KO-f primers, respectively, followed by sequencing using the same primers. To test the ability to respond to exogenous SilCR, the indicated strains were transformed with p*P_4_-gfp* and induction of GFP production was measured in the presence of 10 µg ml^−1^ SilCR as described in “Materials and Methods”. ND - not determined.
(0.04 MB DOC)

Figure S1 The dependence of P_4_ activity on SilCR concentration in the GAS JS95 and GGS N9 strains. To calculate promoter activity, JS95 (A) or N9 (B) harboring p*P_4_-gfp* were grown to OD_600_ = 0.2, then SilCR was added to the indicated concentrations and the cultures were further incubated for 1 h. Fluorescence intensities were determined at time 0 and then every 15 min as described in “Materials and Methods”. The slopes of fluorescence intensity as a function of time (representing initial rates of GFP accumulation) were calculated by performing least square analyses which yielded coefficients of determination greater than 0.95 (R^2^>0.95) and depicted here as promoter activity. The values shown are the mean±the standard deviation results of at least two independent experiments.
(0.04 MB JPG)

Figure S2 The *silE-blpM* intergenic region. The sequence between *silE* and *blpM* start codons (red letters and arrows) is shown. DR1 and DR2 sequences are marked in blue. Transcription start sites are indicated by yellow circles [12]. Primers used for construction of p*P_3_-gfp* and p*P_4_-gfp* are indicated by black bended arrows. The location of exonuclease III digestion stop points, described in Figure 2D and in “Materials and Methods”, are pointed by numbered rectangles.
(0.10 MB JPG)

Figure S3 Analysis of the transcript initiated from the P6 promoter (A) Transcript analysis. Chromosomal DNA and cDNA of bacteria grown to OD_600_ = 0.3 in the presence of 10 µg ml^−1^ of SilCR were prepared as described in “Materials and Methods”. M represents the DNA marker “Hyper Ladder 1” (Bioline, UK). The PCR products shown in lanes 1–5 were produced by subjecting either DNA or cDNA samples to PCR reactions, using the sets of primers specified and detailed in Table S3. RNA was used as a template in lane 5* to demonstrate that there was no contamination of DNA prior to cDNA synthesis. (B) Effect of SilCR on the transcriptional activation of genes regulated by the P_6_ promoter. Transcript amounts of the *blpU* and the three downstream genes (*ORF2-4*) relative to that of *gyrA* were measured by real-time RT-PCR for the JS95 strain grown with or without 10 µg ml^−1^ SilCR to OD_600_ = 0.3. To demonstrate that the upregulation in the transcription is dependent on the SilA-SilB TCS, we also measured the transcript of *blpU* in the JS95Δ*silAB* mutant, which was grown in the presence and absence of SilCR as described above. The values shown are the mean±the standard deviation of at least two independent experiments.
(0.08 MB JPG)

Figure S4 Blocking of SilCR-mediated P_4_ activation by anti-SilCR antibody. Anti-SilCR antiserum was added to JS95p*P_4_-gfp* culture medium at serial dilutions of 100 and 1000-fold before the addition of SilCR. Cultures containing 0, 10 and 100 ng ml^−1^ SilCR were then grown to an OD_600_ = 0.3 and the relative fluorescence was determined as described in “Materials and Methods”.
(0.05 MB JPG)
