# Supplementary material for: Functional Analysis of the Quorum-Sensing Streptococcal Invasion Locus (sil)
Source: PLoS Pathog. 2009 Nov 6;5(11):e1000651. doi: 10.1371/journal.ppat.1000651 (PMC2766830; doi:10.1371/journal.ppat.1000651)
Supplement: Table S1 — Strains (0.07 MB DOC) [file ppat.1000651.s001.doc]

**Table S1. Strains:**

| **Strain** | **Relevant genotype/description** | **Reference** |
| --- | --- | --- |
| ***E. coli*** |  |  |
| JM109 | A cloning strain. | Promega |
| **GAS** |  |  |
| JS95 | M14-type isolated from a NF patient. | [11] |
| JS95Δ*silAB* | *silAB* deletion mutant of JS95. | This study |
| JRS4 | Spontaneous streptomycin resistant derivative of WT strain D471 of M6-type. | [56] |
| IB7 | M29-type isolated from an infected sinus. Israeli Ministry of Health. | This study |
| IB7Δ*silAB* | *silAB* deletion mutant of IB7. | This study |
| IB7Δ*silCR* | *silCR* deletion mutant of IB7. | This study |
| T6 | M102-type, throat isolate (#17315). Israeli Ministry of Health. | This study |
| T13 | M118-type, throat isolate (#18337). Israeli Ministry of Health. | This study |
| T14 | M29-type, throat isolate (#18589). Israeli Ministry of Health. | This study |
| T25 | M4-type, throat isolate (#18750). Israeli Ministry of Health. | This study |
| NS27 | M41-type, wound isolate. | [15] |
| NS40 | M type not identified, wound isolate. | This study |
| NS42 | M15-type isolated from a NF patient. | [15] |
| NS47 | M4-type, wound isolate. | [15] |
| NS48 | M41-type, wound isolate. | [15] |
| NS75 | M69-type, wound isolate. | [15] |
| NS84 | M3-type, wound isolate. | [15] |
| NS86 | M41-type, abscess isolate. | [15] |
| **GGS** |  |  |
| N3 | Wound isolate (#V13616), Germany. | This study |
| N3*silE^-^* | *silE* insertion-inactivated derivative of N3 | This study |
| N4 | Wound isolate (#V00174), Germany. | This study |
| N7 | Blood isolate (#V01618), Germany. | This study |
| N9 | Knee joint isolate (#V13790), Germany. | This study |
| N10 | Tonsil isolate (#V00068), Germany. | This study |
| NS79^1^ | Abscess isolate. | [15] |
| NS88^1^ | Wound isolate. | [15] |
| Z1 | Throat isolate (#20901), Israeli Ministry of Health. | This study |
| Z14 | Throat isolate (#18755), Israeli Ministry of Health. | This study |
| Z19 | Throat isolate (#16849), Israeli Ministry of Health. | This study |
| Z35 | Throat isolate (#17400), Israeli Ministry of Health. | This study |
| Z45 | Throat isolate (#21992), Israeli Ministry of Health. | This study |

^1^ These strains were previously reported as GAS but were found to be GGS by the latex agglutination test for grouping of beta-hemolytic streptococci.
