# Supplementary material for: Functional Analysis of the Quorum-Sensing Streptococcal Invasion Locus (sil)
Source: PLoS Pathog. 2009 Nov 6;5(11):e1000651. doi: 10.1371/journal.ppat.1000651 (PMC2766830; doi:10.1371/journal.ppat.1000651)
Supplement: Table S2 — Plasmids (0.04 MB DOC) [file ppat.1000651.s002.doc]

**Table S2. Plasmids:**

| **Plasmid** | **Description** | **Reference** |
| --- | --- | --- |
| pGEM-T-Easy | A commercial T-vector for AT cloning | Promega |
| pG*silAB* | pGEM-T-Easy derived plasmid containing *silA* and *silB* genes | This study |
| pG*silAB-*ΩKm | pG*silAB*-derived plasmid containing an ΩKm resistance cassette flanked by ~500 bp fragments of *silA* and *silB* | This study |
| pJRS233 | Streptococcus-E. coli temperature sensitive shuttle vector | [51] |
| pJ*silAB-*ΩKm | pJRS233 derived plasmid containing an ΩKm resistance cassette flanked by ~500 bp fragments of *silA* and *silB* | This study |
| pKSM 410 | *Streptococcus*-*E. coli* shuttle vector harboring a promoterless *gfp* | [52] |
| p*P_3_-gfp* | pKSM 410 derived plasmid containing a 233 bp region located upstream to *silE* (contains both DR1 and DR2) | This study |
| p*P_4_-gfp* | pKSM 410 derived plasmid containing a 154 bp region located upstream to *blpM* (contains only DR2) | This study |
| p*P_4_-gfp* *silAB* | p*P4-gfp* derived plasmid containing *silA* and *silB* together with P_1_ and a transcriptional terminator | This study |
| pJ*silE^-^* | pJRS233 derived plasmid containing a 635 bp internal fragment of *silE* | [6] |
