# Supplementary material for: Functional Analysis of the Quorum-Sensing Streptococcal Invasion Locus (sil)
Source: PLoS Pathog. 2009 Nov 6;5(11):e1000651. doi: 10.1371/journal.ppat.1000651 (PMC2766830; doi:10.1371/journal.ppat.1000651)
Supplement: Table S3 — Primers (0.07 MB DOC) [file ppat.1000651.s003.doc]

**Table S3. Primers:**

| **Primer name** | **Sequence** |
| --- | --- |
| General primers ^1^ |  |
| SP-02 | ATCTGCCACAAAGACTGATCAAG |
| SP-04 | GGAGTTGGTTTATCAAATGTCAG |
| *silA-*f | ATTAGCTAGCCATCACCATCACCATCACAATATTTTTGTCTTAG |
| *silB*-r $%$0 0 | GTCACGATCGCATTCATAAAAGCTTCCAAGAGTCT |
| *silEp*-f (*NsiI*)$ % $0  0silEp-r (StuI)$ % $0 | CTT*ATGCAT*AAATACTAAGCTATTATG |
| *silEp*-r (*StuI*)$ % $0  0blpMp-f (NsiI)$ % $0 | AG*AGGCCT*ATATCATAAAGAACAGTA |
| *blpMp*-f (*NsiI*)$ % $0  0blpMp-r (XhoI)$ % $0 | CTT*ATGCAT*ATCATTTAAACTTCTTAAT |
| *blpMp*-r (*XhoI*)$ % $0  0  silAp-f$Xh 0 silB- | AAC*CTCGAG*CTAGTACAGAATA |
| *silAp*-f$Xh 0 silB-TT-r$  0gfp-r$-r 0sra-f$c- 0sra-r$c 0 silE-04-r$ | TTCAGATGAGGTGGTTTAAGC |
| *silB*-TT-r | TCATTCATAAAAGCTTCCAAGAGTCTTT |
| *gfp*-r$-r 0sra-f$c- 0sra-r$c 0 sil | GTTGCATCACCTTCACCC |
| FAM-*spec*-r | TAGTCCACTCTCAACTCCTGA |
| *sra*-f$c- 0sra-r$c 0 silE-04-r$s0 | CTGATGCTACTGCCATAGCAG |
| *sra*-r$c 0 silE-04-r$s0 | GCGTTCAGGAAGTCTAGCTC |
| ABC-KO-f | ATGACACTTGTTACACGTCC |
| *silE*-04-r | TATCAAACAGAGCTTTCTGATGG |
| Point mutations primers ^2^ |  |
| PM-10bp spacer-f | GACATTTCATGATGAAAAACGGCCTTTTAAG |
| PM-10bp spacer-r | CTTAAAAGGCCGTTTTTCATCATGAAATGTC |
| PM-12bp spacer-f | GACATTTCATGATGAA**C**AAAACGGCCTTTTAAG |
| PM-12bp spacer-r | CTTAAAAGGCCGTTTT**G**TTCATCATGAAATGTC |
| PM-A1C-f | GTGTAGAGAGGATAAAAAG**C**CATTTCATGATGAAAAAACGGC |
| PM-A1C-r | GCCGTTTTTTCATCATGAAATG**G**CTTTTTATCCTCTCTACAC |
| PM-C2G-f | GTGTAGAGAGGATAAAAAGA**G**ATTTCATGATGAAAAAACGGC |
| PM-C2G-r | GCCGTTTTTTCATCATGAAAT**C**TCTTTTTATCCTCTCTACAC |
| PM-T4G-f | GTAGAGAGGATAAAAAGACA**G**TTCATGATGAAAAAACGGCC |
| PM-T4G-r | GGCCGTTTTTTCATCATGAC**C**TGTCTTTTTATCCTCTCTAC |
| PM-T5C-f | GTAGAGAGGATAAAAAGACAT**G**TCATGATGAAAAAACGGCC |
| PM-T5C-r | GGCCGTTTTTTCATCATGA**C**ATGTCTTTTTATCCTCTCTAC |
| PM-T6C-f | GTAGAGAGGATAAAAAGACATT**C**CATGATGAAAAAACGGCC |
| PM-T6C-r | GGCCGTTTTTTCATCATG**G**AATGTCTTTTTATCCTCTCTAC |
| PM-C7G-f | GTAGAGAGGATAAAAAGACATTT**G**ATGATGAAAAAACGGCC |
| PM-C7G-r | GGCCGTTTTTTCATCAT**C**AAATGTCTTTTTATCCTCTCTAC |
| PM-A8C-f | GTAGAGAGGATAAAAAGACATTTC**C**TGATGAAAAAACGGCC |
| PM-A8C-r | GGCCGTTTTTTCATCA**G**GAAATGTCTTTTTATCCTCTCTAC |
| PM-T9C-f | GTAGAGAGGATAAAAAGACATTTCA**C**GATGAAAAAACGGCC |
| PM-T9C-r | GGCCGTTTTTTCATC**G**TGAAATGTCTTTTTATCCTCTCTAC |
| PM-G10C-f | GTAGAGAGGATAAAAAGACATTTCAT**C**ATGAA |
| PM-G10C-r | GGCCGTTTTTTCAT**G**ATGAAATGTCTTTTTAT |
| Real Time RT-PCR primers |  |
| R-*blpA*-f | TTACTTTAACGGAGGGATCT |
| R-*blpA*-r | TGCTGCTCTAGCTTCGATT |
| R-*blpU*-f | GGATGAGGCCATGCTTACT |
| R-*blpU*-r | AACAAGTCGCCCCATAAC |
| R-ORF2-f | CGACTGTAGTTTCTCAACCA |
| R-ORF2-r | CTTGTTGATAAGCCATTCC |
| R-ORF3-f | TTGCTCCTTCACCAACAT |
| R-ORF3-r | ACCATTACACGCATTAGG |
| R-ORF4-f | AGTGGGTACGTGATGCTTT |
| R-ORF4-r | CCTGTTGCACCAATCTTT |
| R-*gyrA*-f | CGACTTGTCTGAACGCCAAA |
| R-*gyrA*-r | TTATCACGTTCCAAACCAGTCAA |

^1^ Underlined italic sequences represent recognition sites of restriction endonucleases.

^2^ Underlined sequences indicate the first repeat of DR2 and the nucleotides in bold show the position of the point mutation.
