# Supplementary material for: Functional Analysis of the Quorum-Sensing Streptococcal Invasion Locus (sil)
Source: PLoS Pathog. 2009 Nov 6;5(11):e1000651. doi: 10.1371/journal.ppat.1000651 (PMC2766830; doi:10.1371/journal.ppat.1000651)
Supplement: Table S4 — Predicted SilA binding sites and their genetic context (0.05 MB DOC) [file ppat.1000651.s004.doc]

**Table S4. Predicted SilA binding sites and their genetic context.**

| **Genomic location (5')** | **Score** | **P-value** | **Sequence** | **Binding site strand** | **Gene strand** | **Gene name** | **Gene symbol** | **Gene product** |
| --- | --- | --- | --- | --- | --- | --- | --- | --- |
| 404654 | -8.60 | 0.E+00 | ACCATTCATGCTTTTATAAATACCTTTTAAG | - | - | *silE* | *spy_0408* | Bacteriocin ABC transporter |
| 404713 | -8.60 | 0.E+00 | ACATTTCATGATGAAAAAACGGCCTTTTAAG | + | + | *blpM* | *spy_0409* | Bacteriocin |
| 394624 | -11.72 | 0.E+00 | ACCATTCAGGATATACTAGATACCATTTAAG | - | - | *blpA* | *spy_0395* | Bacteriocin ABC transporter |
| 396215 | -12.69 | 0.E+00 | ACCATTCAAGATGTTTCGATGTCCGTTTAAG | - | + | *ORF2* | *spy_0397* | Bacteriocin-like protein |
| 394699 | -13.22 | 0.E+00 | TCATTTCATGATGAAAAAAGAACATTTGAAG | + | + | *blpU* | *spy_0396* | Bacteriocin |
| 542926 | -15.43 | 0.E+00 | CCTATTATAGGAATAGACGAGACCTTTTTAG | - | + | *asnC* | *spy_0556* | Asparaginyl-tRNA synthetase |
| 214100 | -16.56 | 1.E-06 | ACATTTTAGGACGAAAAAGCAACATTTCAGG | + | + | ** fasX* |  | Regulatory RNA |
| 1485090 | -16.90 | 3.E-06 | ACCATTAGGGTGGGATTAGACTCTTTTTAGG | - | - | *-* | *spy_1552* | Enoyl- reductase (NADH) |
| 405608 | -16.95 | 3.E-06 | TCCATTTATGATAGTCAGACCATTTTTTTAG | - | + | *spy0484* | *spy_0410* | Hyp. membrane associated protein |
| 601947 | -17.12 | 3.E-06 | TCCTCTAATGGTAACACATACACCTCTTTAG | - | + | *rplS* | *spy_0634* | 50S ribosomal protein L19 |
| 958103 | -17.13 | 3.E-06 | ACTAATTATGTTTGTTACGCAACAATTAAAG | - | - | *apbE* | *spy_1009* | Iron-sulfur repair protein ApbE |
| 935727 | -17.42 | 5.E-06 | ATCATTATTGTACAAAAATACACGATTTAAG | - | - | *-* | *spy_0985* | Zn-dependent alcohol dehydrogenase |
| 1140949 | -17.78 | 9.E-06 | TCCTTTATTGATTCATTAATCACCCGTTATG | + | - | *-* | *spy_1195* | Thioesterase superfamily protein |
| 980108 | -17.81 | 8.E-06 | AAAAATTCTGCTAAAAACAGAACTTTTTAAG | - | + | *rnhB* | *spy_1032* | Ribonuclease HII |
| 1013469 | -17.96 | 1.E-05 | TCTTTTAATGTTTATTCTTTAACTGTTTTCG | + | - | *-* | *spy_1069* | Peptidoglycan lysine |
| 1461614 | -18.02 | 1.E-05 | ACTTTTATTTCGAAGCACTAAACTTTTTTAG | - | - | *nusA* | *spy_1525* | Transcription elongation factor NusA |
| 1587386 | -18.04 | 1.E-05 | ACATTTATGGTATAGTCTAAATCAAATTAAG | - | - | *-* | *spy_1651* | Peptidase family M22 |

All the positions are based on the M4 genome (NC_008024). The genes located downstream to putative SilA binding sites are described. When no gene annotations were available, a BLASTP search was performed as described in "Materials and Methods".

* *fasX* is a putative regulatory RNA, which does not appear in the genome annotation but was reported previously [57].
