# Supplementary material for: Functional Analysis of the Quorum-Sensing Streptococcal Invasion Locus (sil)
Source: PLoS Pathog. 2009 Nov 6;5(11):e1000651. doi: 10.1371/journal.ppat.1000651 (PMC2766830; doi:10.1371/journal.ppat.1000651)
Supplement: Table S5 — Microarray analysis (0.10 MB DOC) [file ppat.1000651.s005.doc]

**Table S5. Microarray analysis^1^**:

| **Gene symbol** ^2^ | **Gene name / function** ^3^ | **Functional classification** | **WT, 10 μg ml^-1^ SilCR, 180 min** ^4^ | | **WT, 10 μg ml^-1^ SilCR, 10 min** | | **WT, 0.05 μg ml^-1^ SilCR, 10 min** | | **Δ*silAB*, 10 μg ml^-1^  SilCR, 10 min** | |
| --- | --- | --- | --- | --- | --- | --- | --- | --- | --- | --- |
|  |  |  | fold of change | P-value | fold of change | P-value | fold of change | P-value | fold of change | P-value |
| Spy0031 | *purE* | metabolism | 2.45 | 0.02 | 1.01 | 0.53 | 0.93 | 0.80 | 1.04 | 0.00 |
| Spy0032 | *purK* | metabolism | 4.84 | 0.01 | 1.06 | 0.76 | 1.11 | 0.79 | 0.98 | 0.01 |
| Spy0036 | *plcR* | gene regulatory function | 2.25 | 0.00 | 0.90 | 0.82 | 0.80 | 0.92 | 0.86 | 0.00 |
| Spy0099 | *comG* 6 | competence related | 0.48 | 0.04 | 0.79 | 0.96 | 0.90 | 0.47 | 0.82 | 0.63 |
| Spy0150 | Hyp.^5^ protein | unknown function | 2.54 | 0.03 |  |  | 3.26 | 0.00 | 0.80 | 0.00 |
| Spy0152 | *metB* | metabolism | 2.13 | 0.01 | 0.87 | 0.78 | 0.88 | 0.50 | 0.93 | 0.09 |
| Spy0322 | Hyp. export protein | transport protein | 2.48 | 0.02 | 0.88 | 0.83 | 1.36 | 0.97 | 1.28 | 0.28 |
| Spy0347 | *metS* | metabolism | 2.90 | 0.01 | 1.09 | 0.88 | 1.64 | 0.74 | 1.06 | 0.92 |
| Spy0388 | *dgk* | gene regulatory function | 2.08 | 0.04 |  |  | 0.94 | 0.94 |  |  |
| Spy0395 | *blpA* | *sil* | 4.15 | 0.00 | 1.74 | 0.09 | 1.35 | 0.17 | 1.01 | 0.04 |
| Spy0398 | *blpU* | *sil* | 19.24 | 0.01 | 19.08 | 0.00 | 9.13 | 0.01 | 1.36 | 0.87 |
| Spy0399 | Hyp. protein (ORF4) | *sil* | 20.55 | 0.01 | 17.91 | 0.09 | 13.28 | 0.12 | 1.32 | 0.69 |
| Spy0402 | *IS904A* | *sil* | 1.82 | 0.00 | 2.26 | 0.00 | 1.44 | 0.00 | 1.17 | 0.41 |
| Spy0404 | *silB* | *sil* | 5.53 | 0.00 | 1.45 | 0.00 | 1.23 | 0.00 | 1.12 | 0.55 |
| Spy0405 | *silCR* | *sil* | 13.77 | 0.01 | 9.57 | 0.00 | 5.68 | 0.00 | 1.22 | 0.56 |
| Spy0407 | *silD* | *sil* | 12.36 | 0.00 | 11.61 | 0.00 | 13.10 | 0.00 | 0.88 | 0.01 |
| Spy0409 | *blpM* | *sil* | 12.73 | 0.01 | 9.67 | 0.00 | 10.35 | 0.01 | 1.07 | 0.09 |
| Spy0411 | Bacteriocin-like peptide | *sil* | 13.70 | 0.00 | 8.80 | 0.00 | 7.45 | 0.02 | 1.06 | 0.66 |
| Spy0416 | Hyp. histidine kinase | gene regulatory function |  |  | 2.98 | 0.00 | 3.16 | 0.01 | 0.89 | 0.61 |
| Spy0420 | *mutR* | gene regulatory function | 3.53 | 0.04 | 1.08 | 0.95 | 1.16 | 0.59 | 0.73 | 0.64 |
| Spy0520 | Permease component | transport protein | 0.43 | 0.00 | 0.99 | 0.81 | 1.37 | 0.25 | 0.91 | 0.33 |
| Spy0644 | *sagA* | virulence factor | 2.17 | 0.04 |  |  | 1.05 | 0.76 |  |  |
| Spy0677 | Hyp. ABC transporter | transport protein | 0.44 | 0.00 | 1.10 | 0.64 | 0.85 | 0.62 | 1.09 | 0.41 |
| Spy0689 | *rmlD* | metabolism | 2.37 | 0.03 | 0.84 | 0.85 | 0.90 | 0.64 | 1.00 | 0.49 |
| Spy0712 | *aroF* | metabolism | 2.03 | 0.04 |  |  |  |  |  |  |
| Spy0808 | Hyp. protein | unknown function | 2.13 | 0.05 | 0.81 | 0.78 | 0.94 | 0.52 | 0.84 | 0.56 |
| Spy0813 | Glutathione S-transferase | metabolism | 2.23 | 0.00 | 1.19 | 0.19 | 0.62 | 0.23 | 0.82 | 0.30 |
| Spy0930 | Hyp.l protein | unknown function | 2.28 | 0.03 | 0.87 | 0.59 | 0.75 | 0.75 | 1.04 | 0.00 |
| Spy0931 | *ptsA* | metabolism | 8.40 | 0.01 | 1.10 | 0.80 | 1.35 | 0.31 | 0.79 | 0.47 |
| Spy0932 | *ptsB* | metabolism | 5.44 | 0.02 | 1.16 | 0.84 | 0.86 | 0.96 | 0.71 | 0.15 |
| Spy0933 | *ptsC* | metabolism | 3.80 | 0.00 | 1.35 | 0.71 | 1.46 | 0.95 | 1.53 | 0.64 |
| Spy0934 | *ptsD* | metabolism | 2.18 | 0.02 | 0.83 | 0.75 | 0.94 | 0.58 | 1.07 | 0.87 |
| Spy1061 | *pknB*-like | gene regulatory function | 2.60 | 0.04 | 0.92 | 0.93 | 0.80 | 0.82 | 1.37 | 0.84 |
| Spy1063 | Phage repressor | gene regulatory function | 2.28 | 0.02 | 1.04 | 0.57 | 0.86 | 0.98 | 0.97 | 0.92 |
| Spy1142 | Hyp. ABC transporter | transport protein | 2.13 | 0.04 |  |  | 0.95 | 0.53 |  |  |
| Spy1195 | Thioesterase superfamily | metabolism | 0.44 | 0.00 | 1.00 | 0.49 | 1.05 | 0.41 | 0.83 | 0.82 |
| Spy1242 | Permease MFS superfamily | transport protein | 4.40 | 0.00 | 0.96 | 0.68 | 0.97 | 0.65 | 0.87 | 0.09 |
| Spy1244 | *coiA*-like | competence related | 3.22 | 0.04 | 1.13 | 0.69 | 0.85 | 0.88 | 1.48 | 0.28 |
| Spy1344 | *artP* | transport protein | 2.81 | 0.02 | 0.94 | 0.87 | 1.13 | 0.90 | 0.72 | 0.64 |
| Spy1496 | *glyQ* | metabolism | 3.12 | 0.01 | 1.04 | 0.92 | 0.65 | 0.58 | 1.14 | 0.07 |
| Spy1557 | *dnaJ* | chaperone | 2.05 | 0.01 |  |  |  |  |  |  |
| Spy1598 | *scrK* | metabolism | 2.44 | 0.01 | 1.11 | 0.70 | 0.76 | 0.56 | 1.05 | 0.79 |
| Spy1601 | *scrA* | metabolism | 2.88 | 0.02 | 0.85 | 0.73 | 0.94 | 0.95 | 1.16 | 0.79 |
| Spy1639 | MerR family | gene regulatory function | 2.52 | 0.02 | 0.80 | 0.87 | 1.04 | 0.60 | 0.86 | 0.14 |
| Spy1804 | Hyp. protein | unknown function | 2.53 | 0.01 | 0.94 | 0.81 | 0.88 | 0.47 | 0.94 | 0.03 |
| Spy1881 | *nrdG* | metabolism | 2.84 | 0.04 |  |  | 0.77 | 0.74 | 1.01 | 0.32 |
| Spy1882 | Acetyltransferase | metabolism | 2.18 | 0.00 |  |  | 0.94 | 0.21 | 1.30 | 0.77 |
| Spy1884 | 30S ribosomal protein | metabolism | 2.08 | 0.03 | 0.97 | 0.47 | 1.04 | 0.84 | 0.76 | 0.28 |

^1^ Fold of change in gene transcription in the presence of SilCR and the P-values were calculated as described in "Materials and Methods". The empty cells represent results where the replicate probes were highly inconsistent.

^2^ Based on M4-type genome ([NC_008024](http://www.ncbi.nlm.nih.gov/nuccore/NC_008024)).

^3^ When no annotations were available, BLASTP or BLASTN searches were performed as described in "Materials and Methods".

^4^ Two biological replicates were performed and the average values are presented.

^5^ Hyp. stands for hypothetical.
