# Supplementary material for: Functional Analysis of the Quorum-Sensing Streptococcal Invasion Locus (sil)
Source: PLoS Pathog. 2009 Nov 6;5(11):e1000651. doi: 10.1371/journal.ppat.1000651 (PMC2766830; doi:10.1371/journal.ppat.1000651)
Supplement: Table S6 — Characterization of sil in GGS isolates. sil presence was verified by PCR using the SP-02 and SP-04 primers. The silCR start codon and the intactness of silD were tested by amplifying the corresponding regions by PCR using the SP-02 and SP-04, and silE-04-r and ABC-KO-f primers, respectively, followed by sequencing using the same primers. To test the ability to respond to exogenous SilCR, the indicated strains were transformed with pP4-gfp and induction of GFP production was measured in the presence of 10 µg ml−1 SilCR as described in “Materials and Methods”. ND - not determined. (0.04 MB DOC) [file ppat.1000651.s006.doc]

**Table S6. Characterization of *sil* in GGS isolates**

| **Strain** | ***sil*** | ***silCR* start codon** | ***silD*** | **Response to SilCR** |
| --- | --- | --- | --- | --- |
| N3 | + | ATG | Intact | + |
| N4 | + | ATG | Truncated | ND |
| N7 | + | ATG | Truncated | - |
| N9 | + | ATG | Truncated | + |
| N10 | + | ATG | Truncated | ND |
| NS79 | + | ATG | ND | ND |
| NS88 | + | ATG | ND | ND |
| Z1 | + | ND | ND | + |
| Z14 | + | ND | ND | + |
| Z19 | + | ND | ND | - |
| Z35 | + | ND | ND | + |
| Z45 | + | ND | ND | - |

*sil* presence was verified by PCR using the SP-02 and SP-04 primers. The *silCR* start codon and the intactness of *silD* were tested by amplifying the corresponding regions by PCR using the SP-02 and SP-04, and *silE*-04-r and ABC-KO-f primers, respectively, followed by sequencing using the same primers. To test the ability to respond to exogenous SilCR, the indicated strains were transformed with p*P_4_-gfp* and induction of GFP production was measured in the presence of 10 µg ml^-1^ SilCR as described in "Materials and Methods". ND – not determined.
