# Supplementary figures and images for: Functional Analysis of the Quorum-Sensing Streptococcal Invasion Locus (sil)
Source: PLoS Pathog. 2009 Nov 6;5(11):e1000651. doi: 10.1371/journal.ppat.1000651 (PMC2766830; doi:10.1371/journal.ppat.1000651)

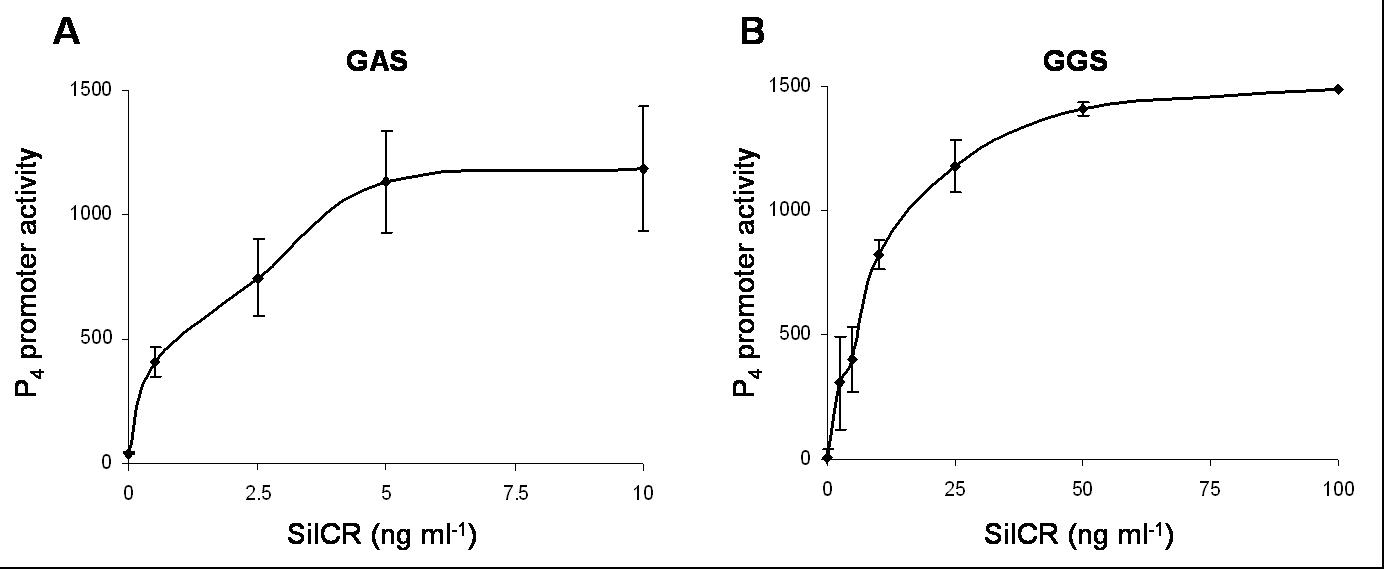

Supplement: Figure S1 — The dependence of P4 activity on SilCR concentration in the GAS JS95 and GGS N9 strains. To calculate promoter activity, JS95 (A) or N9 (B) harboring pP4-gfp were grown to OD600 = 0.2, then SilCR was added to the indicated concentrations and the cultures were further incubated for 1 h. Fluorescence intensities were determined at time 0 and then every 15 min as described in “Materials and Methods”. The slopes of fluorescence intensity as a function of time (representing initial rates of GFP accumulation) were calculated by performing least square analyses which yielded coefficients of determination greater than 0.95 (R2>0.95) and depicted here as promoter activity. The values shown are the mean±the standard deviation results of at least two independent experiments. (0.04 MB JPG) [file ppat.1000651.s007.jpg]

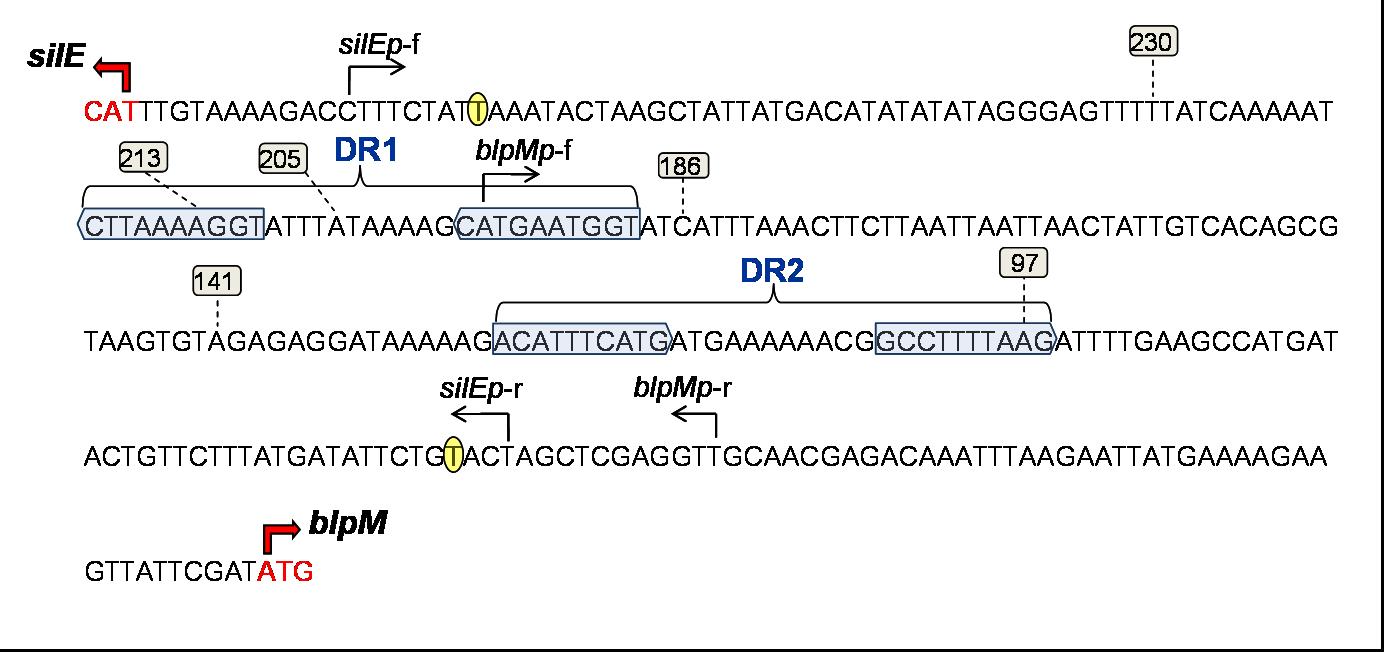

Supplement: Figure S2 — The silE-blpM intergenic region. The sequence between silE and blpM start codons (red letters and arrows) is shown. DR1 and DR2 sequences are marked in blue. Transcription start sites are indicated by yellow circles [12]. Primers used for construction of pP3-gfp and pP4-gfp are indicated by black bended arrows. The location of exonuclease III digestion stop points, described in Figure 2D and in “Materials and Methods”, are pointed by numbered rectangles. (0.10 MB JPG) [file ppat.1000651.s008.jpg]

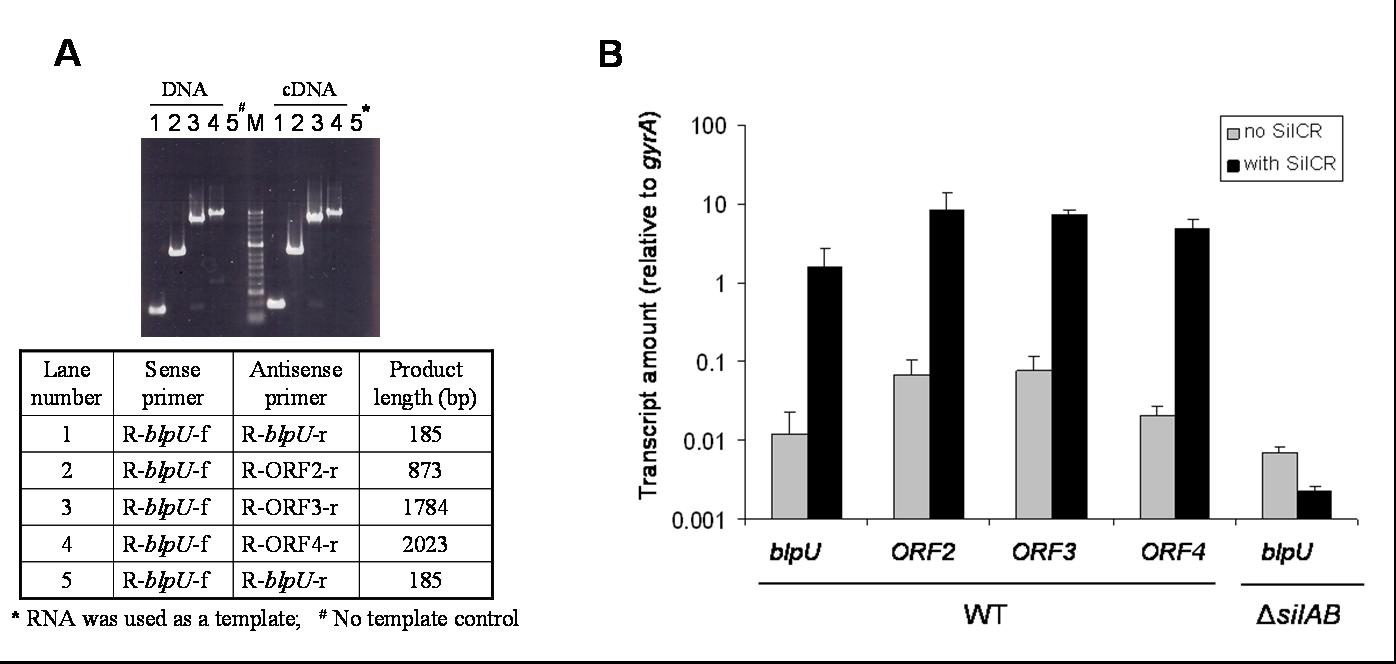

Supplement: Figure S3 — Analysis of the transcript initiated from the P6 promoter (A) Transcript analysis. Chromosomal DNA and cDNA of bacteria grown to OD600 = 0.3 in the presence of 10 µg ml−1 of SilCR were prepared as described in “Materials and Methods”. M represents the DNA marker “Hyper Ladder 1” (Bioline, UK). The PCR products shown in lanes 1–5 were produced by subjecting either DNA or cDNA samples to PCR reactions, using the sets of primers specified and detailed in Table S3. RNA was used as a template in lane 5* to demonstrate that there was no contamination of DNA prior to cDNA synthesis. (B) Effect of SilCR on the transcriptional activation of genes regulated by the P6 promoter. Transcript amounts of the blpU and the three downstream genes (ORF2-4) relative to that of gyrA were measured by real-time RT-PCR for the JS95 strain grown with or without 10 µg ml−1 SilCR to OD600 = 0.3. To demonstrate that the upregulation in the transcription is dependent on the SilA-SilB TCS, we also measured the transcript of blpU in the JS95ΔsilAB mutant, which was grown in the presence and absence of SilCR as described above. The values shown are the mean±the standard deviation of at least two independent experiments. (0.08 MB JPG) [file ppat.1000651.s009.jpg]

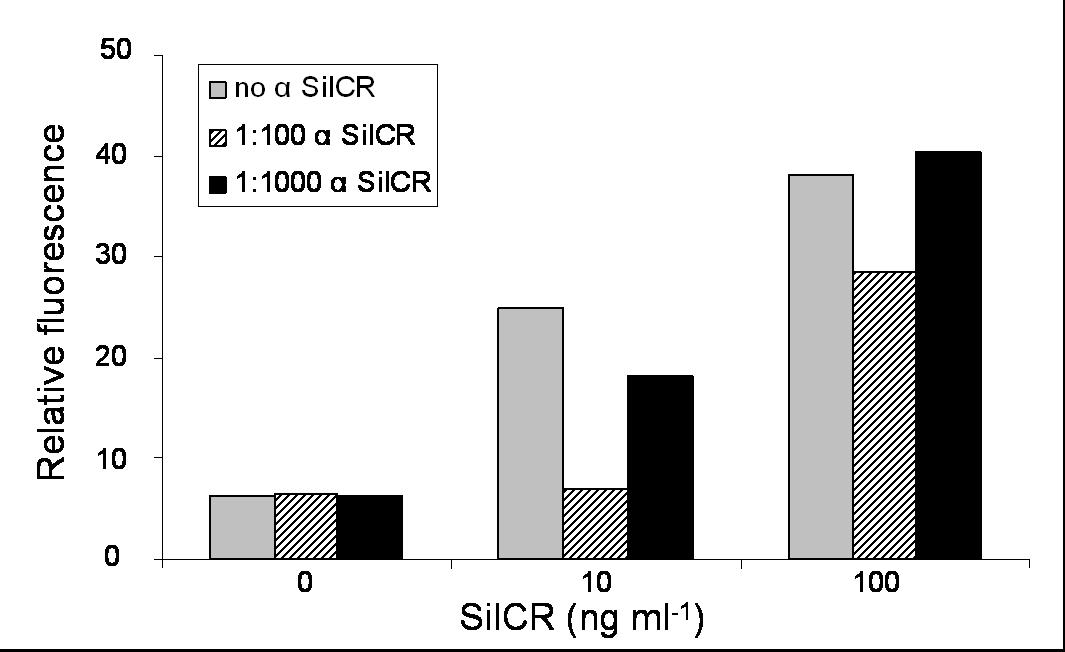

Supplement: Figure S4 — Blocking of SilCR-mediated P4 activation by anti-SilCR antibody. Anti-SilCR antiserum was added to JS95pP4-gfp culture medium at serial dilutions of 100 and 1000-fold before the addition of SilCR. Cultures containing 0, 10 and 100 ng ml−1 SilCR were then grown to an OD600 = 0.3 and the relative fluorescence was determined as described in “Materials and Methods”. (0.05 MB JPG) [file ppat.1000651.s010.jpg]
